# Small-fiber degeneration, sensory dysfunction, and nociception comorbidity in central neurodegenerative disease

**DOI:** 10.3389/fnagi.2026.1854989

**Published:** 2026-10-21

**Authors:** Will Hauser, Jessica E. Keller, Mamatha Pasnoor, Douglas E. Wright

**Affiliations:** 1 Department of Anesthesiology, The University of Kansas Medical Center, Kansas City, KS, United States; 2 Department of Neurology, The University of Kansas Medical Center, Kansas City, KS, United States

**Keywords:** Alzheimer's disease, Amyotrophic Lateral Sclerosis (ALS), nociception, pain, Parkinson's disease, peripheral nervous system, sensory dysfunction, small fiber neuropathy (SFN)

## Abstract

Progressive neurodegenerative disease typically calls for clinical effort to improve cognitive and motor deficits. It is also focused on improving patients' quality of life. However, across these dementias and other diseases, the underlying causes and potential mechanisms remain unclear. Most experimental and clinical research focuses on the central nervous system, examining pathogenic proteins such as tau and amyloid beta, as well as the potential cascade following mitochondrial damage and neuroinflammation. Although peripheral involvement, including gut microbiome influences and other visceral organs, is well-documented, the peripheral nervous system remains an understudied component of these progressive diseases. As peripheral terminals of primary afferent neurons are exposed to inflammatory or toxic mediators, these neurons respond to these threats via neuron-specific phenotypic changes, including the generation of pain. Although normal peripheral sensitization is protective, exposure to damage in a chronic disease state can have long-term detrimental effects. Diseases that lead to central neurodegeneration often also feature peripheral neuropathy and chronic pain. We provide an overview of the incidence of peripheral neuropathy within these diseases, the contribution of changes in sensation to disease, and secondary neuropathic phenotypes resulting from disease. Woven in are discussions of the intersection of degeneration and toxic protein aggregation, briefly touching on resulting inflammatory signaling. Altogether, this review aims to synthesize current understanding of sensory peripheral nerve involvement across these neurodegenerative diseases.

## Introduction

Pain and sensory disturbances are significant, yet often underappreciated, components of neurodegenerative diseases such as Parkinson's disease, Alzheimer's disease, and amyotrophic lateral sclerosis. These conditions involve progressive motor or cognitive decline, but new clinical and preclinical evidence includes altered nociception, small-fiber neuropathy, and chronic pain syndromes within these disorders. These peripheral nerve complications strongly affect a patient's quality of life, but the biological mechanisms underlying these sensory manifestations remain poorly understood. Underlying pathogenic proteins also exert deleterious effects on the peripheral nervous system (PNS), as preclinical studies now show these proteins modulate pain pathways and peripheral nerve integrity, suggesting a broader, more systemic pathophysiological process.

The goal of this review is to highlight PNS involvement in neurodegenerative diseases that affect millions all over the world. Although these neurodegenerative diseases differ, they share several common features, including abnormal protein accumulation, neuroinflammation, metabolic changes, mitochondrial dysfunction, and cognitive decline. Most of the focus is on central nervous system (CNS) mechanisms, leaving a gap in investigations of PNS involvement. These neurodegenerative conditions exhibit variable pathologies associated with increased motor dysfunction; however, sensory dysfunction is surprisingly evident. This review explores the current understanding of pain and peripheral neuropathy in neurodegenerative conditions, highlighting clinical evidence of these complications in patients. We also briefly discuss the contributions of tau, Aβ, α-synuclein, TDP-43, and other pathogenic proteins across various diseases. Our goal is to identify co-morbidities of small-fiber peripheral neuropathy, sensory abnormalities and nociception, and dementia-associated central neurodegeneration. Ideally, this can allow further research of common mechanisms between peripheral and central degeneration and broaden future therapeutic strategies to include altered peripheral sensation in neurodegenerative disease.

## Peripheral neuropathy

### Overview

Peripheral neuropathy affects more than 20 million people in the United States and involves damage to peripheral nerves (Alkandari and Hollywood, 2023). The prevalence of peripheral neuropathy has been reported in up to 7% of the general population, with older adults more often affected (Castelli et al., 2020). Peripheral neuropathies can affect any peripheral nerve, and sometimes also affect the spinal roots, ganglia, autonomic nerves, cranial nerves, or nerve trunks (Hammi and Yeung, 2025). Neuropathies can often be classified into motor, sensory, autonomic, or a combination. Patients often display some combination of these affected fiber types.

### Clinical distribution patterns

Clinically, neuropathies are classified as a mononeuropathy, polyneuropathy, or multifocal neuropath (Hanewinckel et al., 2016b). Mononeuropathies involve entrapment or nerve trauma, whereas polyneuropathies are generally associated with systemic complications (Misra et al., 2008). Polyneuropathies typically affect multiple peripheral nerves, while mononeuropathies usually occur with symptoms at a certain site, then spread to a different, asymmetric site. Mononeuropathy differs from polyneuropathy in that the entire nerve trunk is affected, whereas in polyneuropathy, the primary targets are axons or myelin (Marchettini et al., 2006). Acute sensory neuropathy involves damage to a single nerve or a specific group of nerves, and typically resolves fairly quickly (Ginsberg, 2020). However, persistent inflammation and vascular changes in sensory DRG may drive chronicity (Windebank et al., 1990). Polyneuropathy, whether acute or chronic, commonly presents as distal and symmetrical sensory symptoms that involve a length-dependent pattern (Mirian et al., 2023). Neuropathies can also be non-length-dependent and lack the traditional stocking-glove distribution (Gemignani et al., 2022).

### Etiology and risk factors

The most common cause of generalized peripheral neuropathy is diabetes, as up to 60% of patients with type 2 diabetes subjects had various forms of neuropathy (Dyck et al., 1993). Meta-analyses have shown that neuropathy is less prevalent in patients with type 1 diabetes compared to type 2 diabetes (Sun et al., 2020). Type 1 diabetic neuropathy is seen in around 6% at the onset of Type 1 Diabetes (Sempere-Bigorra et al., 2021), and according to one study, it presents in up to 30% of these patients (Martin et al., 2014). Prediabetes is an important step in diabetes progression, and the detection of neuropathy is increasing. Studies suggest that neuropathy in prediabetes is variable but can be detected in 10%−70% of patients with prediabetes (Kirthi et al., 2021).

Peripheral neuropathy is also prevalent in autoimmune diseases such as Sjögren syndrome, cancers, infections, inherited disorders, vitamin deficiencies, and hypothyroidism (Gwathmey, 2016; Azhary et al., 2010). In addition, obesity appears to be an important risk factor, as polyneuropathy was common in overweight individuals but had normal glycemic control (Callaghan et al., 2020). Other common peripheral neuropathies include chronic demyelinating polyneuropathy (CIDP) (Querol et al., 2017) and chemotherapy-induced peripheral neuropathy (CIPN) (Geber et al., 2013). CIDP is an autoimmune disorder that attacks the myelin sheath, leading to pain and degeneration, and autoantibody treatments are commonly used to address CIDP. CIPN often results from chemotherapeutic agents damaging peripheral nerves, also leading to degeneration and pain.

### Age-associated peripheral nerve changes

It is important to acknowledge that general aging can have large influence on both functional and morphological features of the PNS. Many studies have reported both loss of unmyelinated and myelinated nerves in elderly people. Axonal atrophy also commonly occurs in aged nerves, thought to be due to reduced axonal transport of important cargo and nutrients. Additionally, there is prominent age-related decline in response to injury, as the interaction between Schwann cells and regenerative processes takes much longer (Verdú et al., 2000) In one cross-sectional population based study, 4,179 participants, ranging from ages 41–96 years old, who did not meet the criteria for polyneuropathy were screened for neurological examination, nerve conduction velocity, and questionnaire. It was found that normal features of neurological examination and SNAP amplitude declined with age (Taams et al., 2023). Another study of persons aged from 2 months to 87 years used EMG to determine significant lengthening of CMAP latency with age (Voitenkov et al., 2017). Finally, a meta-analysis performed identified that IENFD decreased with aging by 1.35 fibers/mm every 5 years (Samolis et al., 2025).

### Motor neuropathy

Pure motor neuropathies, in which efferent motor fibers innervating muscle are typically affected, leading to weakness and impaired coordination (Verschueren, 2017). Multifocal motor neuropathy is a rare condition characterized by progressive muscle weakness affecting multiple motor nerves throughout the body without sensory involvement, likely due to chronic inflammation (Hameed and Cascella, 2025). Besides the rare multifocal phenotype, many motor neuron diseases, such as Amyotrophic Lateral Sclerosis (ALS), feature prominent motor neuropathy (Foesleitner et al., 2023).

### Large fiber neuropathy

Sensory ataxia due to loss of joint position sense, loss of vibratory sense are manifestations of large-fiber neuropathy, in which large afferents, such as low-threshold mechanoreceptors and proprioceptors, are more affected (Misra et al., 2008). Large-fiber neuropathy can be diagnosed from nerve conduction studies, where the focus on the function of myelin is important. Large-fiber neuropathy is seen in central neurodegenerative diseases. In one study of Parkinson's Disease, nerve conduction studies of the sural and peroneal nerves were performed in nearly 50% of PD-diagnosed subjects, with abnormal results commonly including reduced compound muscle action potentials (de Araújo et al., 2016). In a study of Alzheimer's Disease, peripheral nerve conduction velocity analysis revealed that the median motor nerve amplitude was lower in AD subjects than in controls, and that common peroneal nerve velocities significantly correlated with AD diagnosis (Qian et al., 2022). Another study of ALS patients noted pathological changes in ascending sensory fibers in 60% of patients with ALS, and subclinical sensory defects in 85% of study participants (Iglesias et al., 2015). For the rest of this review, we will focus on small-fiber neuropathy and the implications of this co-morbidity within central neurodegenerative disease.

### Small-fiber neuropathy

Small-fiber neuropathies involving small, unmyelinated nerves that innervate the epidermis are commonly seen clinically. Clinical consequences include, but are not restricted to, distal-predominant sensory loss and pain (Gomatos et al., 2025). Damage to these nerves can manifest as symptomatic gain or loss of function (Lopes et al., 2022). Gain-of-function symptoms include heightened pain sensitivity, burning, or tingling (paresthesia) (Marchettini et al., 2006), whereas loss-of-function symptoms lead to hyposensitivity or complete loss of sensation (numbness) (Feldman et al., 2019; Javed et al., 2015). Small-fiber neuropathies are normally classified into sensory and autonomic. Small-nerve fibers are either thinly myelinated Aδ fibers or unmyelinated C fibers, and they detect pain and temperature, as well as serve in autonomic function. Mechanical, thermal, and chemical stimuli can activate these receptors, and the fibers can undergo plasticity in response to both their environment and pathogen-associated molecular patterns commonly seen in progressive disease (Lowy et al., 2021). Sensory nociception occurs when these fibers have abnormal responses to noxious stimuli, such as allodynia, where a stimulus that normally does not provoke pain causes reactivity, or hyperalgesia, where noxious stimuli are more painful than usual. This can happen in both cold and heat receptors, as well as mechanical and autonomic fibers (Lacomis, 2002).

### Sex differences in small-fiber neuropathy

There appear to be no clear-cut sex differences in small-fiber neuropathy. Clinical studies have shown that SFN is more common in men (Pál et al., 2020). Another study looking at alcohol-related small-fiber neuropathy found significantly greater frequency in males than females (Zambelis et al., 2005). Other studies have shown little to no difference, emphasizing that males do not have reduced intraepidermal nerve fiber density (IENFD) compared to females (Weaver et al., 2018). Other risk factor studies emphasize that males are more likely to suffer from peripheral neuropathy, even in the absence of disease such as diabetes (Hicks et al., 2021). Interestingly, while neuropathy may be clinically less extreme in females, they often report more intense resulting pain (Abraham et al., 2018).

### Diagnosis of small-fiber neuropathy

Peripheral neuropathy is significantly underrecognized, as it is estimated that nearly 80% of people with neuropathy remain undiagnosed (Gad et al., 2024). Most of the patients with small fiber neuropathy present with distal symmetric presentation, however other distributions can be seen and diagnosis can be difficult in some cases (Devigili et al., 2020). Current diagnosis of peripheral neuropathy requires patient evaluation, history, and rigorous clinical assessment (Castelli et al., 2020; Azhary et al., 2010), electrodiagnostic testing and laboratory testing including vitamin B12, blood glucose, serum immunofixation electrophoresis inflammatory markers, thyroid-stimulating hormone, and metabolic profile (Lehmann et al., 2020; England et al., 2009). Despite all the testing about 25%−46% may not have an identifiable cause and are considered idiopathic. Recent literature shows that metabolic syndrome is also associated with increased risk of neuropathy (Kazamel et al., 2021). Diagnosis may also include skin biopsy, autonomic testing (England et al., 2009), and other electrophysiological analyses (Castelli et al., 2020; Hanewinckel et al., 2016a; Farhad et al., 2016). An important clinical approach for studying small-fiber peripheral neuropathy is quantitative analysis of IENFD, which provides insight regarding peripheral nerve health. Quantitative assessment using corneal confocal microscopy can also be used, as increasing studies reveal that SFN can be inferred from corneal nerve fiber length and density (Jin et al., 2021). Abnormal IENFD in distal regions (ankle) is one of the best indicators of SFN, particularly when paired with qualitative sensory testing (QST) (Egenolf et al., 2021). As part of the ACTTION criteria for diagnosing small-fiber idiopathic neuropathy, there must be at least one small-fiber clinical symptom, abnormal IENFD, and normal sensory NCS (Freeman et al., 2020). Ongoing efforts to standardize and improve clinical diagnoses and management are ongoing (Atmaca et al., 2024).

### Relevance to central neurodegenerative disease

Peripheral insults can affect central neuronal function, as neuroinflammatory and metabolic changes can send signals up to the brain. Additionally, if peripheral nerves are damaged, altered signal may be sent up to the brain, and the central nervous system might be alerted that there is a threat that needs responded to. While motor changes as well as large peripheral fiber abnormalities are commonly seen in central neurodegenerative diseases, the incidence and contribution of small-fiber neuroapthy is less known. A recent review highlighted more than 70 human diseases or conditions with comorbid epidermal axon loss, as shown by IENFD measurements. Neurodegenerative diseases were prominent in this analysis, with comparatively severe reductions in IENFD in these studies of patients with neurodegenerative disease (Thomas et al., 2023). These findings shed new light on the peripheral nerve involvement in neurodegenerative disease. Sensory neuropathies within central neurodegenerative diseases call attention to an underappreciated component of peripheral pathology within disease progression. Below, we highlight the incidence of small-fiber peripheral neuropathy in traditional neurodegenerative disease. We also outline symptoms such as sensory loss throughout different sensory modalities and broader incidences of pain, but with a primary focus on peripheral nociception. Lastly, we also briefly intertwine evidence of pathogenic proteins in sensory neurons within these diseases that point to similarities in central and peripheral pathology.

## Parkinson's disease

### Disease overview

Parkinson's disease (PD) is a progressive neurodegenerative disorder, highlighted by motor dysfunction such as tremor, rigidity, and bradykinesia (Moustafa et al., 2016). There are an estimated 1 million people in the United States living with PD, and there is a significantly increased prevalence with age. The incidence of PD is expected to rise by over 30% by 2030, posing increasing healthcare and economic challenges (Kouli et al., 2018).

PD is a complex disorder that involves interconnected pathological mechanisms. A key component of PD is the excessive accumulation of α-synuclein, a component of Lewy bodies encoded by the *SNCA* gene (Table 1). It transitions from its normally unfolded or tetrameric state into β-sheet-rich structures that are prone to aggregation (Meade et al., 2019). Aggregation of α-synuclein is central to PD development, but it is not the only mechanism underlying PD onset and progression. Mitochondrial dysfunction, impaired protein clearance, and neuroinflammation are significant contributors to PD. PD is also influenced by both genetic and environmental factors (Pizarro-Galleguillos et al., 2023). The SNCA gene is also known as PARK-1, and it harbors mutations linked to α-synuclein accumulation (Zafar and Yaddanapudi, 2025). Over 20 monogenic forms and 100+ risk loci for PARK-1 have been identified since then, with key monogenic forms including SNCA (PARK1), PRKN (Parkin, PARK2), LRRK2 (PARK8), and GBA (Jankovic and Tan, 2020). PARK-1 is only one of the multiple genes linked to PD. Idiopathic PD can also occur in the absence of known gene mutations, with evidence that both tau and Aβ proteins act synergistically with α-synuclein to enhance neuroinflammation during disease progression (Webster et al., 2025) (Table 1).

Disease-modifying therapies are currently limited for PD. Immunotherapies, protein clearance strategies, genetic therapies, and GLP-1 receptor agonists are used to mitigate symptoms. Treatments are also aimed at replacing dopamine or mimicking its effects, with levodopa (L-DOPA) as the most common approach. These interventions manage symptoms but do not halt disease progression. A major obstacle to advancing neuroprotective treatments is the absence of reliable, sensitive biomarkers to track disease progression (Jankovic and Tan, 2020).

As briefly mentioned before, PD is typically thought of as a motor disorder with resulting bradykinesia, tremor, rigidity, gait, and reduced grip force (Moustafa et al., 2016). Non-motor symptoms, such as fatigue, constipation, sleep disturbances, and depression, are less-discussed, but occur in PD and can precede motor symptoms not only by years, but also by decades (Kouli et al., 2018). Although the timeline is less clear, non-motor symptoms, such as pain and sensory dysfunction, can also precede motor symptoms. These non-motor symptoms significantly affect patients' quality of life, and damage to the sensory nervous system is an underappreciated complication of PD.

### Abnormalities in sensation in PD

Sensory disturbances—including paresthesia, itching, visual impairment, and olfactory dysfunction—affect up to 90% of patients with PD (Koller, 1984; Peña-Zelayeta et al., 2025) (Table 1). A longitudinal study assessing functional and morphological changes in cutaneous nerves in patients with PD found that 30% reported sensory symptoms (Nolano et al., 2018). There is also a high prevalence of pain in PD, reported to be 40%−85% of patients according to one study (Beiske et al., 2009; Mostofi et al., 2021) (Table 1). It is clear that “chronic pain is underreported in PD” (Nègre-Pagès et al., 2008). Varied pain phenotypes have been reported, with musculoskeletal pain, chronic widespread pain, and neuropathic pain being the most common, as well as orofacial and dystonic pain (Thomas et al., 2024; Verhoeff et al., 2022) (Figure 1). Pain is more commonly reported as burning, itching, numbness, and tingling, indicating sensory nociception (Djaldetti et al., 2004; Defazio et al., 2008). In one study of 20 individuals with PD and nociceptive pain, it was found that subjects had both lower warm detection and mechanical detection, indicative of hyperalgesia (Barboza et al., 2023) (Figure 1). Additionally, mechanical allodynia has been observed in a study conducted by Touch Neurology that tested wearable technology in PD patients, as well as in another study specifically looking at sensory nerve fiber function in PD (Figure 1). While sensory hypersensitivity is often observed, even in the off state of levodopa use in PD (Vela et al., 2012), increased thermal threshold is also seen, indicative of hyposensitivity (Pressnell et al., 2025; Krämer et al., 2021) (Figure 1). It is proposed that disruptions in peripheral nociception or alterations in central pain thresholds or processing contribute to pain in PD (Tai and Lin, 2020).

### Small-fiber neuropathy in PD

#### Sensory

The prevalence of clinically-diagnosed neuropathy ranges from roughly 5%−55% in PD patients (Corrà et al., 2023), compared to the general population at 2%−8% (Hammi and Yeung, 2025). In a study that used comprehensive clinical, neurophysiological, and neuropathological evaluations, neuropathy was assessed with symptom-based questionnaires (Neuropathy Impairment Score for Lower Limbs [NIS-LL]) and the modified Toronto Clinical Neuropathy Score (mTCNS). Using the NIS-LL, 36.3% of PD-diagnosed subjects showed signs of peripheral neuropathy, whereas 12% reported symptoms based on mTCNS (Corrà et al., 2023). Two indicators of peripheral neurodegeneration have been identified in PD: (Alkandari and Hollywood, 2023) a significant length-dependent loss of intraepidermal small nerve fibers, and (Castelli et al., 2020) a marked, non-length-dependent reduction in substance P–immunoreactive intraepidermal fibers (Doppler et al., 2014). Small-fiber neuropathy is common, as one study showed a significant loss of IENF in PD patients compared with controls. Abnormal sensory function, along with IENF loss, all correlated with disease length and severity, with greater changes in patients with PD for longer than 96 months (Nolano et al., 2018). Significant sensory IENFD reduction has been reported in various other studies (Lama et al., 2022; Jeziorska et al., 2019b). Additional clinical studies suggest that QST revealed that over 21.2% of study participants had impaired sensitivity to cold and heat pain (Corrà et al., 2023). Although neuropathy appears to worsen with disease length, it appears that neuropathy can be detected in early-stage PD (Ramachandran et al., 2022; Jeziorska et al., 2019a). Reduced epidermal nerve innervation in patients with PD has been reported in both the proximal and distal legs compared with normative values (Corrà et al., 2023). Other studies evaluating IENFD found reductions in somatic measures in PD patients compared with controls (Liu et al., 2025). Additionally, corneal nerve fiber density has also been to be significantly reduced in PD patients (Kass-Iliyya et al., 2015a).

It's important to point out that peripheral neuropathy has been positively correlated with levodopa treatment in PD (Toth et al., 2008; Mancini et al., 2014). These studies suggested that polyneuropathy was positively correlated with PD disease duration but also with levodopa treatment doses (Ramachandran et al., 2022). Moreover, in both the ON and OFF states of levodopa treatment, neuropathy, predominantly small-fiber neuropathy (70% of tested subjects) was significantly associated with shorter stride length, slower gait speed, and smaller toe-off angles during straight walking (Corrà et al., 2023). It's clear that neuropathy in PD patients is complex, and awareness of treatment effects is required. However, in idiopathic PD, this presumed secondary complication of peripheral neuropathy can occur in the absence of levodopa treatment as well (Ramachandran et al., 2022).

#### Autonomic

Autonomic small-fiber neuropathy is also seen in PD patients. In one study, significant IENFD loss that was found at the distal leg in 80% of subjects was correlated with sudomotor volumes at the foot, as well as with UPDRS and CASS (Novak) (Table 1). In another study, SWT was found to be abnormal in 57.6% of the 38 PD patients tested (de Araújo et al., 2016). Finally, in a study that used a neuropathy symptom inventory questionnaire, they found that responses from PD patients indicating small-fiber neuropathy were significantly correlated with autonomic dysfunction (Xie et al., 2025).

### Peripheral alpha-synuclein protein in PD

The accumulation of misfolded α-synuclein in PD commonly occurs in the substantia nigra, leading to dopaminergic neuronal loss in the basal ganglia and motor impairment. α-synuclein aggregation has been hypothesized to initiate in the enteric nervous system, with transmission to the brain via the vagus nerve. This is believed to account for early non-motor symptoms such as constipation and REM sleep disorders that precede motor symptoms by years, or often by decades (Zafar and Yaddanapudi, 2025). There is evidence of α-synuclein generation in the gastrointestinal tract, and misfolded α-synuclein produced in the gut can be transmitted to the brain via the vagus nerve (Xu and Pu, 2016). Multiple studies have shown that phospho-α-synuclein deposits are evident in somatosensory nerve fibers of the proximal thigh and distal leg skin (Melli et al., 2018; Donadio et al., 2014; Rodríguez-Leyva et al., 2016; Vacchi et al., 2021, 2022) (Table 1). These studies commonly report an association between skin denervation and α-synuclein deposits, with one study reporting that over 30% of PD subjects with small fiber neuropathy have phospho-α-synuclein pathology in peripheral nerves (Corrà et al., 2023). Another study detected phosphorylated α-synuclein in somatosensory nerve fibers in 50% of patients with PD, compared with none in control patients.

#### Summary

PD is a common progressive neurodegenerative disorder characterized by motor and non-motor symptoms. Pathologically, PD is characterized by widespread α-synuclein accumulation, particularly in neuronal Lewy Bodies, distinguishing it from related disorders such as MSA. Sensory abnormalities and pain are highly prevalent, affecting up to 90% of patients according to discussed studies, with evidence of altered thermal and mechanical sensitivity and small-fiber neuropathy. Small-fiber degeneration, both sensory and autonomic is very common and clinically significant, likely contributing to worse motor outcomes. Further exploration and therapeutic interventions targeting the PNS may prove beneficial in slowing disease progression.

**Table 1:** Summary of sensory dysfunction, pain, and small-fiber neuropathy in PD.

Parkinson's disease	
Primary pathologic protein (s)	Alpha synuclein
Prevalence of sensory disturbances	Up to 90%
Prevalence of pain	40%−85%
Prevalence of small-fiber neuropathy	Up to 80%

Prevalence of disturbances (such as loss of smell, loss of hearing, loss of feeling to touch), pain, and small-fiber peripheral neuropathy. Sensory abnormalities and small-fiber degeneration may be linked to the pathologic proteins underlying PD. Prevalences are based on findings from several publications discussed in the text.

## Amyotrophic lateral sclerosis

### Disease overview

Amyotrophic Lateral Sclerosis (ALS) is a rare, progressive neurodegenerative disease that was previously thought of as a pure motor neuron disease, but is now recognized as a systemic, multifactorial disorder. ALS affects about 9.1 per 100,000 people in the United States (Mehta et al., 2023). The clinical presentations of classic ALS include typical spinal onset, bulbar onset, flail arm syndrome, flail leg phenotype, hemiplegic variant, pseudo-polyneuritic type, mixed forms, and thoracic or respiratory onset variants (Quinn and Elman, 2020). Executive function is the most affected cognitive domain in ALS, and cognitive and/or behavioral changes affect nearly 50% of patients with ALS (Ilieva et al., 2023). Motor neuron death is a key hallmark, but neuromuscular disconnection and TDP-43 protein aggregates serve as biomarkers and are present in up to 95% of patients (Masrori and Van Damme, 2020).

A key breakthrough in ALS research was the identification of pathological aggregates of the TDP-43 protein (Table 2). Present in ~97% of sporadic ALS and most familial ALS cases (Ilieva et al., 2023), TDP-43 inclusions are found in motor cortex projection neurons and somatomotor neurons of the brainstem/spinal cord (Jo et al., 2020). Studies suggest phosphorylated-TDP-43 aggregates propagate sequentially via axonal transport, particularly along corticospinal tracts (Jo et al., 2020). Over 50 unique genes are implicated in ALS. Although TDP-43 mutations are rare, their occurrence appears to impair RNA processing. Other ALS-related mutations include genes encoding SOD1, c9orf72, FUS, and TARDBP (Nijs and Van Damme, 2024). Whether familial or sporadic, ALS includes pathophysiological changes such as neuroinflammation, glutamate-mediated excitotoxicity, mitochondrial dysfunction, and hypermetabolism (Bradford and Rodgers, 2024).

Due to its complexity, ALS has shifted its view toward improved treatment and prognosis. Recent advances include improved understanding of phenotypic heterogeneity, new diagnostic criteria, recognition of biomarkers and modifiable risk factors, development of predictive tools, and the emergence of mechanism-based therapies (Feldman et al., 2022). Currently, three main FDA-approved drugs for ALS can slow down progression: Riluzole, Edaravone, and Tofersen (Ansari et al., 2024). Other treatment approaches include supplements, diet, and speech therapy, all aimed at improving quality of life. Respiratory support is also common, as respiratory failure is one of the leading causes of death in ALS (Mehta et al., 2023).

While motor dysfunction is typically associated with ALS, non-motor symptoms can significantly impact quality of life but are often overlooked and undertreated. The most frequently reported symptoms include urinary urgency, limb pain with coldness, changes in smell/taste, and loss of appetite—all more prevalent in ALS than in control subjects (Shojaie et al., 2024). Though some of these symptoms also occur in the general population, they are significantly more prevalent in people with ALS and may serve as early disease markers or predictors of progression and treatment response. Autonomic disturbances, pain, and psychiatric symptoms are common complications of ALS (Chowdhury et al., 2021). Leg swelling, fatigue, muscle cramps, and sensory symptoms, including tingling, have also been reported despite normal nerve conduction studies (Shojaie et al., 2024). These non-motor symptoms strongly impact quality of life in ALS but often are not defined or consistently included in clinical assessments.

### Abnormalities in sensation in ALS

Sensory dysfunction is an under-recognized but important aspect of motor diseases such as ALS, affecting patient care and offering insight into disease spread and subcortical involvement. Sensory nervous system deficits have been reported in up to 30% of ALS patients (Table 2) (Seki et al., 2023), and paresthesia, pain, and taste changes have been reported in ALS, pointing to sensory involvement (Kleinerova et al., 2025). Additionally, patients exhibit increased sensitivity to external stimuli (Kleinerova et al., 2025; Liu et al., 2019). Somatosensory pathway degeneration is evident at multiple levels: cortical, subcortical, and white matter tracts. Sensory dysfunction is commonly reported in patients with ALS, as sensory tracts of the visual, olfactory, and gustatory systems are additionally affected in ALS (Rubio et al., 2022). Changes in sensation are not limited to these systems or the somatosensory cortex; abnormalities can occur at the spinal level and at distal sites (Table 2). The visual and auditory systems exhibit structural changes even in the absence of overt symptoms. In addition to cerebellar dysfunction, proprioceptive deficits likely contribute to gait and balance impairments and falls (Chipika et al., 2022).

Chronic pain is a common but often overlooked symptom in ALS. Pain is reported throughout the disease course and contributes to reduced quality of life, depression, and worse clinical outcomes, especially in later stages. However, patients with ALS often fail to report pain (Handy et al., 2011). Primary pain in ALS can be nociceptive, neuropathic, nociplastic, or due to muscle cramps, with varied causes and locations (Chiò et al., 2017). Orofacial pain has also been reported (Pang and Park, 2015) (Figure 1). In contrast, secondary pain is mainly nociceptive and is associated with muscle weakness and atrophy, prolonged immobility leading to joint and connective tissue degeneration, and extended use of home mechanical ventilation (Kwak, 2022) (Figure 1). It's been reported that proximal upper and distal lower limbs were more frequently affected in patients with painful ALS (Taga et al., 2019). Sensory nociception is also widely prevalent in ALS. Between 50%−78% of ALS patients experience pain, with about one third of these patients describing the pain as “electric” or “burning” (Rubio et al., 2022; Chiò et al., 2017) (Figure 1), with presentations of both hyperalgesia, allodynia, and hypoesthesia possible (Table 2). One clinical study reported that 35% of ALS-diagnosed patients reported sensory symptoms, with both large-fiber (e.g., paresthesia) and small-fiber (e.g., burning pain, allodynia, reduced pain or temperature sensation) symptoms. Burning pain was the most observed small-fiber symptom, and sensory complaints increased significantly across King's clinical stages of ALS, especially for small-fiber symptoms (Nolano et al., 2024). Importantly, increased pain and sensory deficits should raise alarms about the sensory degeneration in ALS.

### Small-fiber neuropathy in ALS

#### Sensory

Peripheral neuropathy is well-documented within ALS, and traditional polyneuropathy and ALS share similar pathologies. Sensory neuropathy occurs in up to 20% of ALS patients, involving both large and small nerve fibers (Bombaci et al., 2023). A small clinical study of ALS patients found that all subjects had sensory neuropathy that was not due to a separate cause (Isaacs et al., 2007). Early in the ALS progression, axonal degeneration, peripheral axon loss, and nerve terminal destruction occur as key pathological events (Gentile et al., 2019). One study found that IENFD was significantly reduced in ALS patients at the leg, thigh, and fingertip compared to controls, regardless of disease onset type. Interestingly, regenerative attempts (i.e., nerve sprouting) were associated with faster disease progression and poorer ALS-related outcomes (Nolano et al., 2024). Another study took skin biopsies of 28 ALS patients and found significant IENFD in the distal calf compared to controls (Weis et al., 2011). Broadly, significant reductions in IENFD have been reported in numerous other ALS studies (Nolano et al., 2024; Sassone et al., 2016; Isak et al., 2017; Ren et al., 2018). In studies of both sporadic and familial ALS, degeneration of the spinal dorsal column was noted, particularly in lumbar spinal regions (Hammad et al., 2007). Additionally, corneal nerve lengths have been found to be lower in ALS patients (Fu et al., 2022).

ALS type 8 (ALS8) is a familial form of ALS characterized by early lower motor neuron involvement, and clinical studies of patients with ALS8 identified symptom onset at a young age with a longer disease course than sporadic ALS (sALS). Sensory abnormalities were found in 35% of ALS8 and 30% of sALS patients, despite normal sensory nerve action potentials. Reductions in IENFD were significantly greater in ALS8 patients than in healthy controls, and reductions were more pronounced in females (Machado-Costa et al., 2023). Reductions in IENFD were identified in 75% of patients with pure ALS, and IENFD was reduced to a similar degree in patients with bulbar, flail limb, pyramidal, and spinal onset ALS. Surprisingly, however, only 15% of ALS patients in this study reported sensory disturbances, emphasizing the likelihood of underreported peripheral nerve degeneration in ALS (Dalla Bella et al., 2016) (Table 2). Collectively, this evidence suggests that distal sensory small-fiber axon degeneration is a significant, but under reported comorbidity in ALS.

#### Autonomic

Autonomic neuropathy is also seen in ALS. In one study of 86 ALS patients tested, increased composite autonomic severity score was seen in 75% of subjects with mild impairment in 85% and moderate in 15% (Piccione et al., 2015). ALS patients had a significantly higher score of sympathetic, parasympathetic, and overall autonomic dysfunction in one study (Pavlovic et al., 2010). There is also evidence of autonomic skin denervation, with one study showing abnormalities in amyelinic axons and sweat glands in the majority of the 13 ALS patients tested (Provinciali et al., 1994).

## Peripheral TDP-43 and tau protein in ALS

Identification of biomarkers common to ALS, peripheral neuropathy, and other neurodegenerative diseases is consistent with the presence of peripheral neuropathy in ALS. ALS patients show a significant increase in CSF- and blood-based NFL and NFH compared with control groups (Agah et al., 2024). With regard to pTDP-43, small fiber neuropathy was higher in ALS patients who were pTDP-43 positive (Ren et al., 2018). TDP-43 aggregates accumulate within the cell body and axons of sensory neurons (Ayers and Cashman, 2018) (Table 2). This relationship between TDP-43 and peripheral neuropathy in ALS raises a potentially important link between neuropathy and abnormal protein aggregation in neurodegenerative disease. Experimentally, active ALS-linked SARM1 variants promote degeneration and cytotoxicity when expressed in cultured DRG neurons (Bloom et al., 2022). It has also been proposed that peripheral glial cells impact peripheral neuropathy in ALS progression (Philips and Rothstein, 2014), as satellite glial cells in DRG exhibit abnormal SOD1 accumulation (Ruiz-Soto et al., 2020). Growing evidence also suggests elevations of tau and Aβ in the serum and muscles of ALS patients (Abu-Rumeileh et al., 2025). Additionally, postmortem DRG samples from ALS patients have been shown to be almost equal distribution of small molecular weight tau to big tau (44%−55%), implying that forms of tau that are more prone to aggregation are found in DRGs of ALS patients (Koppisetti et al., 2025) (Table 2).

### Summary

ALS is a complex, multifactorial neurodegenerative disease that extends beyond motor neuron degeneration to include systemic and cognitive involvement. Its rapid progression and limited treatment options underscore the urgent need for effective therapies. Importantly, non-motor symptoms—including sensory dysfunction and chronic pain—are prevalent and impact quality of life but remain under-recognized in clinical care. Comprehensive approaches that integrate motor and non-motor features may improve patient outcomes and aid new approaches to treat ALS. Additionally, small-fiber neuropathy is an under reported complication of ALS, and mechanistic studies should be performed to understand why this occurs. Further exploration and therapeutic interventions targeting the PNS may prove beneficial in slowing disease progression.

**Table 2:** Summary of sensory dysfunction, pain, and small-fiber neuropathy in ALS.

Amyotrophic lateral sclerosis	
Primary pathologic protein (s)	TDP-43, Tau
Prevalence of sensory disturbances	Up to 30%
Prevalence of pain	50%−78%
Prevalence of small-fiber neuropathy	Up to 75%

Prevalence of disturbances (such as loss of smell, loss of hearing, loss of feeling to touch), pain, and small-fiber peripheral neuropathy. Sensory abnormalities and small-fiber degeneration may be linked to the pathologic proteins underlying ALS. Prevalences are based on findings from several publications discussed in the text.

## Multiple systems atrophy

### Disease overview

Multiple system atrophy (MSA) is a sporadic and progressive neurodegenerative movement disorder that includes both motor and non-motor symptoms (Krismer et al., 2024). Worldwide MSA is evident in 2–5 per 100,000 people with no apparent sex differences according to one population study done from 2012 to 2021 (Erro et al., 2024). The average survival from symptom onset is 7–9 years, although outcomes vary widely (Goh et al., 2023).

MSA, like PD, features abnormal accumulation of a-synuclein protein, which can either happen through endogenous protein misfolding or template-directed propagation, like other prion proteins (Woerman et al., 2018) (Table 3). Glial cytoplasmic inclusions of a-synuclein are evident in MSA (Watanabe et al., 2023), and α-synuclein inclusions are more evident in oligodendrocytes and Schwann cells in MSA than in Lewy body neural inclusions in PD (Reddy and Dieriks, 2022).

MSA, along with other Atypical Parkinsonism Syndromes (APS), are often misdiagnosed, and early symptoms contribute to this. A postmortem analysis revealed that only 60% of patients with MSA were correctly diagnosed (Koga et al., 2015). Clinical presentation and family history remain the best means of diagnosing MSA. There is a new emphasis on identifying better markers to differentiate MSA from other APS (Kauppila et al., 2022).

Early signs of MSA involve isolated symptoms like autonomic failure, bradykinesia, or cerebellar ataxia (Watanabe et al., 2018). Additional early features include balance impairment, hypotension, urinary problems, dizziness, new-onset snoring, and psychiatric symptoms like depression and mild cognitive impairment (Watanabe et al., 2023). Memory problems are common before diagnosis but less severe than those in other neurodegenerative diseases (Schrag et al., 2007). While motor dysfunction is thought of more frequently in MSA, there is evidence that autonomic dysfunction may even precede it (McKay and Cheshire, 2018). Although early symptoms, often of vast systems, may make diagnosis difficult, it is important to consider morbidities that are not purely motor-related. For example, abnormalities in sensation are underappreciated complications in MSA.

### Abnormalities in sensation in MSA

Changes in sensation are frequently observed in MSA, mostly related to pain. Pain is a frequent complaint from patients with MSA, although pain reporting is still likely underreported in MSA. A meta-analysis estimated a pooled prevalence of pain in MSA of 67%. A year later, this group conducted a community survey of individuals with MSA and caregivers and identified reported pain in 87% of MSA patients (Campese et al., 2024) (Table 3). In another study, 47% of patients with MSA reported pain, and over half identified their pain as sensory related (Tison et al., 1996). Another study reported pain in 78.6% of MSA patients, and pain thresholds were significantly lower than those of PD patients (Ory-Magne et al., 2018). Finally, comparisons of reported pain in patients with either MSA, PD, or progressive supranuclear palsy (PSP) reported that pain was notably worse and more frequent in MSA and PD, particularly in their limbs (Kass-Iliyya et al., 2015b). Different phenotypes of pain in MSA include orofacial (Thongchuam et al., 2020), musculoskeletal (Campese et al., 2023), vascular (Campese et al., 2023), and dystonic (Boesch et al., 2002) (Figure 1). Preclinical studies found changes in thermal and mechanical sensitivity in a PLP-α-syn MSA mouse model, attributed to α-synuclein aggregation in Schwann cells (Figure 1). However, the PLP-α-synuclein MSA mice had no changes in sensory neuron morphology, conduction velocity, or ion channel expression (Kuzdas-Wood et al., 2015). Sensory nociception seems to be a potential co-morbidity of MSA clinically as well. In one study, MSA patients presented impaired cold and heat-sensitivity threshold, impaired thermal sensory limen, and mechanical hyperalgesia, indicating a potential small-fiber neuropathy (Krämer et al., 2021) (Figure 1).

### Small-fiber neuropathy in MSA

#### Sensory

There remains a gap in our understanding of peripheral neurodegeneration in MSA, but it is apparent that it is a complication. Moreover, lower tibial nerve amplitudes were associated with poorer motor outcomes (UMSARS II, UPDRS III), particularly in gait and posture, suggesting that peripheral nerve damage may exacerbate motor disability (Rohmann et al., 2021). These studies raise the question of whether peripheral neuropathy can predict outcomes (Rodolico et al., 2001). Although nerve amplitudes suggest large fiber neuropathy, further studies should be done to see whether small-fiber neuropathy may also exacerbate motor disability. However**,** limited exploration of potential small-fiber degeneration has been reported, and many symptoms likely remain unreported; however, sufficient clinical studies suggest that peripheral neuropathy is highly prevalent in MSA, although specific causes remain unclear (Galassi et al., 1982; Abele et al., 2000). Some studies report that patients with MSA have reduced IENFD, although not to the extent observed in patients with PD (Gibbons et al., 2023). Conversely, other studies have shown that sensory skin denervation was more pronounced in MSA patients than in PD (Nolano et al., 2022). Nevertheless, IENFD has been associated with 1-year clinical disease progression in MSA (ΔUMSARS part II) and seems to be an underrecognized complication of MSA (Andréasson et al., 2025).

#### Autonomic

It has been suggested that the presence of peripheral neuropathy has a stronger predictive value for MSA when autonomic dysfunction is present (Galassi et al., 1982). Sudomotor dysfunction has been associated with baseline measures of disease burden in MSA study participants (Andréasson et al., 2025), suggesting autonomic small-fiber damage. Additionally, autonomic skin denervation is more pronounced in MSA patients than in PD, with 94% of subjects displaying fiber loss (Nolano et al., 2022) (Table 3). Examination of the cornea may benefit analysis of small-fiber neuropathy, as patients with MSA had more severe corneal nerve damage than those with PD, with significant reductions in corneal nerve fiber density and length. These changes in corneal innervation were linked to autonomic impairments (Niu et al., 2024). Although distal nerve degeneration in lower extremities is not as clear, corneal fiber loss in MSA may suggest systemic nerve changes associated with sensory small-fiber neuropathy.

## Peripheral alpha-synuclein protein in MSA

In MSA, α-synuclein deposits are found in only a small portion of noradrenergic fibers in the skin (Donadio et al., 2020, 2023). Neurofilament light chain (NFL) levels in cerebrospinal fluid and blood are reportedly elevated in MSA and correlate with disease severity (Watanabe et al., 2023). It is suggested that MSA patients exhibit stronger peripheral nerve involvement than other APS and are more similar to that in PD, due to shared toxicity from α-synuclein (Rohmann et al., 2021). Although PD patients also have inclusions in Schwann cells, MSA patients have inclusions that are more widely distributed throughout the peripheral nerve (Gibbons et al., 2023) (Table 3). A-synuclein aggregation may contribute to both nociception and small-fiber degeneration in MSA, but further studies need to be performed to support this claim.

### Summary

MSA is a rare, progressive neurodegenerative disorder characterized by motor and non-motor symptoms, including autonomic dysfunction, Parkinsonism, and cerebellar ataxia, with an average survival of under a decade. Pathologically, MSA is characterized by widespread α-synuclein accumulation, particularly in oligodendrocytes and peripheral nerves, distinguishing it from related disorders such as PD. Sensory abnormalities and pain are highly prevalent, affecting up to 67%−87% of patients based on discussed studies, with evidence of altered thermal and mechanical sensitivity and possible small-fiber neuropathy based off of reduced sensory IENFD, skin autonomic fiber denervation, sudomotor dysfunction, and corneal nerve fiber degeneration. Although traditional electrophysiological studies suggest peripheral neuropathy is under-detected, newer evidence indicates it may be more common and clinically significant, contributing to worse motor outcomes. Further exploration and therapeutic interventions targeting the PNS may prove beneficial in slowing disease progression.

**Table 3:** Summary of sensory dysfunction, pain, and small-fiber neuropathy in MSA.

Multiple systems atrophy	
Primary pathologic protein (s)	Alpha Synuclein
Prevalence of sensory disturbances	Up to 87%
Prevalence of pain	Up to 87%
Prevalence of small-fiber neuropathy	Up to 94%

Prevalence of disturbances (such as loss of smell, loss of hearing, loss of feeling to touch), pain, and small-fiber peripheral neuropathy. Sensory abnormalities and small-fiber degeneration may be linked to the pathologic proteins underlying MSA. Prevalences are based on findings from several publications discussed in the text.

## Alzheimer's disease

### Disease overview

Over 55 million people globally and an estimated 7 million Americans aged 65 and older have AD, with an expectation of cases doubling by 2060. Memory loss is the most prominent symptom of AD, but other features include physical decline, difficulty with orientation, and reduced spatial skills. Additionally, there are deficits in communication, judgment and decision-making, swallowing, and behavioral changes.

There are two main types of AD: Familial and Sporadic. FAD accounts for early-onset dementia, with phenotypes resulting from mutations in PSEN1, PSEN2, and APP genes (Cacace et al., 2016). People diagnosed with AD at 65 years and older or with no genetic mutations are classified as sporadic AD, which accounts for 95% of cases (Andrade-Guerrero et al., 2023). Sporadic AD is associated with genetic and environmental factors (Yegambaram et al., 2015), but may also result from inflammatory disease (Aisen and Davis, 1994), autoimmune disease (Ramey et al., 2024), metabolic disease (Liu et al., 2024), and infections (Catumbela et al., 2023). There are two pathological hallmarks of AD: neurofibrillary tau tangles (NFTs), which are intracellular aggregates of abnormally phosphorylated tau protein (Maccioni et al., 2010), and extracellular amyloid plaques composed of the Aβ peptide (Reiss et al., 2018) (Table 4). Both Aβ and tau are also known to interact with α-synuclein, which commonly accumulates in about 50% of AD cases (Shim et al., 2022). Genetic mutations and risk factors also contribute to the accumulation of toxic protein aggregates. APOE4 is an additional risk allele for sporadic AD, affecting Aβ clearance and tau-mediated degeneration (Safieh et al., 2024).

There is growing interest in understanding inflammatory feedback loops and neurodegeneration related to chronic microglial activation in AD (Maccioni et al., 2009; Cortés et al., 2018). It is plausible to suggest that microglial activation reflects some real or perceived cellular “threat” and is triggered as a pro-inflammatory response to various aggregated proteins or neuronal damage. More recent research has shifted toward understanding and targeting inflammation and metabolism, with increased interest in tau pathology as well (Maccioni et al., 2010). Abnormal gait is more common in Familial Alzheimer's Disease (FAD) (Karve et al., 2012). Other non-motor symptoms in AD include diminished sensations and chronic pain. It is important to understand AD as a systemic disease, and expand beyond just end-point memory loss by further understanding all systems involved both during disease and preceding it. One of these systems that is underappreciated in AD is the sensory nervous system.

### Abnormalities in sensation in AD

Abnormalities in sensation are associated with a higher risk of dementia (Brenowitz et al., 2019). Visual, olfactory, and auditory regions typically decline in people with AD. Age-related hearing loss is considered a modifiable risk factor for AD, as studies show defects preceding dementia onset by 5–10 years (Albers et al., 2015). Olfactory dysfunction is common with aging, and studies show significant impairment in up to 90% of AD cases (Table 4) (Devanand et al., 2000). Evidence of retinal ganglion cell atrophy in AD patients, as well as preclinical evidence of retinal fiber degeneration in AD rodent models, both point toward visual impairment in AD (Guo et al., 2010). Additionally, tactile deficits have been seen in AD, specifically a decline in tactile angle discrimination (Yang et al., 2010). Sensory decline in AD prevents interaction with an individual's environment, and these early changes may accelerate the progression of the disease.

Pain is an overlooked symptom of AD. The incidence of pain increases as dementia progresses, but patients are often unable to communicate or interpret it, resulting in poor clinical recognition and treatment. Patients with AD may have changes in facial expression that hide typical visual signs of pain. Orofacial (de Siqueira et al., 2010), musculoskeletal (Tian et al., 2023), muscular (Tian et al., 2023), muscular (Yao et al., 2025), and visceral (Achterberg et al., 2013) are all different types of pain seen in AD (Figure 1). Pain may manifest as aggressive behaviors such as yelling and hitting. Pain can worsen stress, sleep, and fatigue. Chronic pain in AD has a reported prevalence of 45% worldwide (van Kooten et al., 2015) (Table 4), and people with AD-related dementia have been reported to experience significantly more pain than those without (Kumaradev et al., 2021). As a contributor, AD-related neuroinflammation likely worsens pain and aids in dementia progression (Whitlock et al., 2017). Clinical studies report that thermal hyposensitivity and pain were identified in 78% of patients with AD (Table 4) (Figure 1) (Fletcher et al., 2015), implying that sensory nociceptors are also affected. One study of 29 people with mild-to-moderate AD demonstrated significantly lower tolerance to pressure algometry (Jensen-Dahm et al., 2014), indicative of mechanical hyperalgesia (Figure 1). Preclinically, both mechanical allodynia (Cui et al., 2022) and thermal hyperalgesia (Merlo et al., 2023) have also been observed (Figure 1). Combined, this evidence implies that changes in sensory nociceptor function may be an unappreciated comorbidity in AD.

Chronic pain can also increase the risk of AD. A greater number of chronic pain sites are associated with increased risk of AD (Tian et al., 2023). AD-related dementia (ADRD) was significantly higher in patients with previous chronic pain compared to controls, suggesting that chronic pain increased the risk of developing ADRD (Bornier et al., 2023). Moreover, widespread pain was associated with a 47% increase in AD (Wang and Liu, 2021). In fact, multisite pain was found to have a greater dementia risk than diabetes and coronary heart disease (Zhao et al., 2023). It is not clear if pain is causative or a secondary complication of AD, but it likely can serve as both. New approaches need to identify mechanisms that link pain and dementia, including inflammation-related, metabolic dysfunction, and toxic aggregates. Gray matter loss in the hippocampus and prefrontal cortex in patients with chronic pain is consistent with similar changes in patients with AD (Bell et al., 2024; Rodriguez-Raecke et al., 2009). Additionally, similar to AD, chronic pain can affect attention and emotion through dysfunction of the locus coeruleus-noradrenergic (LC-NE) system, and chronic pain hyperactivates the LC-NE system, as in AD (Cao et al., 2019). There could be commonalities in the pathology of the two morbidities.

### Small-fiber neuropathy in AD

Small-fiber degeneration remains poorly explored in AD. It has been reported that a higher warm-detection threshold is associated with AD. In this study, IENFD decreased in the cervical regions but not in the proximal thigh or distal leg (Liu et al., 2025). While it is unclear if small-fiber neuropathy is a secondary complication of AD, peripheral neuropathy correlates significantly with immune activation in patients with dementia (Leblhuber et al., 2011), and the severity of peripheral neuropathy is significantly negatively correlated with cognitive performance (Lin et al., 2021). This suggests that small-fiber neuropathy may be a potential predictor of disease. Additionally, the relationship between ADRD, diabetes, and immune activation is quite strong. There is a higher incidence of ADRD in patients with type 2 diabetes, and patients with diabetic peripheral neuropathy have a higher risk than diabetic patients without peripheral neuropathy (Jin et al., 2023; Mian et al., 2025) (Table 4). More research is needed to understand the relationship between type 2 diabetic PN, aging, and AD.

### Peripheral beta-amyloid and tau protein in AD

Recent studies suggest that these toxic proteins are deposited in the CNS and PNS (Xu et al., 2025; Dugger et al., 2022). Tau and amyloid deposits can be visualized in the CNS via PET scans and also detected in cerebrospinal fluid and skin (Ali et al., 2025; Buchholz et al., 2025). Blood-based biomarkers include ptau-181 and ptau-217, two specific phosphorylated forms of tau that are upregulated in AD, as well as total tau and brain-derived tau (Gonzalez-Ortiz et al., 2024; Sarto et al., 2025). An increased Aβ42/40 ratio is commonly seen (Trelle et al., 2025; Espay et al., 2023; Teunissen et al., 2018). Plasma tau and Aβ have been good biomarkers of AD, offering high sensitivity and specificity for AD diagnosis, but their deposition is not restricted to the brain. One study found pathological tau deposits in multiple peripheral organs, including the stomach, liver, aorta, and spleen, in AD subjects but not in controls (Miklossy et al., 2010).

Preclinical studies demonstrate that phospho-tau is evident in the PNS, including the lumbar DRG (Dugger et al., 2019). Clinical studies have identified several species of phosphorylated tau in peripheral skin (Rodríguez-Leyva et al., 2016) and in skin nerve fibers. In one study, up to 50% of AD subjects had identifiable p-tau in skin nerve fibers, and Aβ1-42 was identified in 30% (Buchholz et al., 2025) (Table 4). Amyloid deposits also appear in dermal papillae (Awal and Kaur, 2018), and there is evidence of amyloid deposits in DRG. This has been extensively studied in amyloid neuropathies, such as transthyretin (TTR) amyloidosis, in which common cutaneous forms of amyloidosis appear more closely related to neurodegenerative amyloidosis (Clos et al., 2012). This suggests that peripheral tau and amyloid species behave similarly in the PNS as in the CNS, becoming pathological agents at similar rates and time points. It is also well-known that Aβ can be cleared from the CNS into the periphery. In fact, Aβ clearance can be modulated by central-peripheral concentration gradients (Marques et al., 2020). It's plausible that peripheral clearance of Aβ in AD may provide benefit as it reduces Aβ burden in the CNS by moving it to peripheral tissues.

Peripheral inflammation is likely a component of pain in patients with dementia, as peripheral inflammation in distal skin correlates with dementia progression (Wen et al., 2022). Given that Aβ and tau aggregates affect both the PNS and CNS, similar pathological mechanisms may underlie comorbidities of skin inflammation, peripheral neuropathy, and pain in patients with dementia, and these may result from a perceived threat to peripheral tissues posed by toxic Aβ or tau aggregates (Schreml et al., 2010).

### Summary

AD is a common, progressive neurodegenerative disorder characterized by dementia, motor, and non-motor symptoms. Pathologically, AD is characterized by widespread tau and Aβ accumulation, particularly in neurons. Sensory abnormalities and pain are highly prevalent, affecting up to 90% of patients, with evidence of altered thermal and mechanical sensitivity and possible small-fiber neuropathy. Although a universally accurate prevalence of peripheral neuropathy is not well-known, small-fiber degeneration seemingly creates a much greater risk for developing AD- related dementia. Further exploration and therapeutic interventions targeting the PNS may prove beneficial in slowing disease progression.

**Table 4:** Summary of sensory dysfunction, pain, and small-fiber neuropathy in AD.

Alzheimer's disease	
Primary pathologic protein (s)	Tau, Amyloid-Beta
Prevalence of sensory disturbances	Up to 90%
Prevalence of pain	45%−78%
Prevalence of small-fiber neuropathy	Diabetic neuropathy is a risk factor

Prevalence of disturbances (such as loss of smell, loss of hearing, loss of feeling to touch), pain, and small-fiber peripheral neuropathy. Sensory abnormalities and small-fiber degeneration may be linked to the pathologic proteins underlying AD. Prevalences are based on findings from several publications discussed in the text.

## Progressive supranuclear palsy

### Disease overview

Progressive supranuclear palsy (PSP) is a rare neurodegenerative disorder that causes dementia and a rapid decline in life expectancy. A recent systematic review of 32 different clinical studies identified an estimated prevalence of PSP to range from 1 to 18 per 100,000 people, and an estimated reported incidence of 0.16–2.6 per 100,000 person-years (Lyons et al., 2023). PSP is a rapidly progressing disease characterized by early axial rigidity, frequent falls, and cognitive decline (Devi et al., 2025). It can be classified as a four-repeat (4R) tauopathy, with abnormal tau phosphorylation and accumulation driving disease phenotypes (Kovacs et al., 2020) (Table 5). Progressive Supranuclear Palsy with Richardson's syndrome (PSP-RS) and Progressive Supranuclear Palsy with predominant Parkinsonism (PSP-P) are both identified subtypes of PSP. Differentiation of the two subtypes is based on clinical evaluation and MRI findings. The two differ in tremor presentations across six midbrain-based parameters, including the classic hummingbird appearance (Wen et al., 2023).

It is generally believed that PSP has multiple causes, including genetic and sporadic contributions. There is limited evidence of sex differences in PSP (Swallow et al., 2022). It is thought that PSP may be more prevalent in males, with some studies also adding that males have an older age of onset but worse progression of the disease (Mahale et al., 2021). PSP commonly begins around age 65, and the median survival is 7–8 years from symptom onset (Testa et al., 2001). Often, diagnosis does not occur for several years (Nysetvold et al., 2024). Clinicians can use the MDS-PSP and NINDS-SPSP guidelines to diagnose PSP, along with imaging and key clinical signs; postmortem analysis is generally required for a definitive diagnosis, as are certain clinical predictors of PSP, such as akinesia, cognitive dysfunction, balance impairment, and ocular-motor issues (Höglinger et al., 2017). 4R tau has been shown to accumulate in brain regions such as the midbrain, basal ganglia, and brainstem. Neuronal loss is very apparent with the presence of NFTs (Debnath et al., 2022). A genome-wide association study identified risk signals for PSP in the STX6, EIF2AK3, and MOBP genes. They also identified two MAPT variants as risk factors for PSP (Höglinger et al., 2017), suggesting that tau pathology in PSP can be initiated in diverse ways. There is suggestive evidence that the precise structure of tau filaments can distinguish among tauopathies (Shi et al., 2021).

PSP affects not only cognition but also movement and balance, vision and speech, and even behavior (Daroff, 1987). Motor symptoms include frequent falls, prominent axial rigidity, dystonia, dysphagia, and bradykinesia. There is also severe frontal lobe dysfunction, often affecting emotional processing (Agarwal and Gilbert, 2025). Nevertheless, clinical characteristics of PSP can be extremely heterogeneous (Respondek et al., 2014). Progressive gait freezing, or failure to initiate gait, is highly predictive of PSP and typically precedes other motor symptoms. This also occurs well before many motor signs and could be associated with sensory nervous system dysfunction (Steele, 1972). No disease-modifying therapies have been identified (Lamb et al., 2016). Therefore, it is important to understand systemic changes and underappreciated co-morbidities to capture the heterogeneity of PSP fully.

### Abnormalities in sensation in PSP

A small clinical study of PSP found that 80% of the 35 PSP subjects examined reported systemic symptoms at baseline (Arena et al., 2016) (Table 5). Tauopathies such as PSP are typically studied in the CNS, but peripheral sensory abnormalities have also been identified. Dysphagia, constipation, and urinary dysfunction have been commonly reported (Baschieri et al., 2023; Oliveira et al., 2019). PSP patients have also shown both altered taste and smell (Chaithra et al., 2020) and gastrointestinal symptoms (Radicati et al., 2017). The Parkinson's Disease non-motor Symptoms (PRIAMO) study concluded that non-motor symptoms were much more common in PSP than in PD or in healthy controls (Colosimo et al., 2010). Pain has also been reported in 11%−68% of patients with PSP (Dubbioso et al., 2021; Stamelou et al., 2012; Schrag et al., 2010) (Table 5). Observed pain phenotypes include dystonic (Barclay and Lang, 1997), visceral (Baschieri et al., 2023), and musculoskeletal (Fang et al., 2021) (Figure 1). There is some evidence of thermal and mechanical hyperalgesia in PSP-RS patients (Stamelou et al., 2012) (Figure 1). Finally, inflammatory signaling in PSP could also be linked to the integrated stress response, which is dysregulated in PSP (Whitney et al., 2024) and is implicated in neuropathic pain (Barragán-Iglesias et al., 2019).

### Small-fiber neuropathy in PSP

#### Sensory

One study found that 66% of PSP subjects presented at least one neuropathic symptom, detailed and quantified by the NSS (Rohmann et al., 2021). Sensory small-fiber neuropathy is an underappreciated complication of PSP. Corneal nerve fiber density has been used to measure peripheral nerve degeneration in patients with PSP. Compared with healthy controls, patients with PSP had significantly lower corneal nerve fiber density (Lim et al., 2023; Woo et al., 2022). In one study, a significant length-dependent loss of sensory fibers compared to controls was found in PSP patients. IENFD was significantly decreased in the legs of PSP patients, with the lower limbs being more severely affected overall. This peripheral nerve degeneration was associated with functional impairment and disease severity. As part of the degeneration, axonal swellings, fiber clusters, and subepidermal nerve sprouting were noted (Dubbioso et al., 2021). Another clinical study reported that 65.2% of the 23 tested PSP subjects had distal symmetric peripheral neuropathy, including significant mixed fiber involvement, compared to PD patients (60.9% of subjects). Surprisingly, polyneuropathy appears to be more common in PSP patients than in PD patients. Interestingly, they also identified an association between disease duration and neuropathy. PSP patients with neuropathy had both a shorter duration of disease and levodopa use (Devi et al., 2025) (Table 5). Evidence of mixed fiber involvement and clear peripheral neuropathy, along with the rapid onset of symptoms, may imply early sensory involvement in PSP. PSP is, however, a more rapidly progressing disease compared to PD (Payan et al., 2011). The association between neuropathy and shorter disease duration may reflect a faster overall rate of disease progression.

#### Autonomic

PSP patients frequently report symptoms suggestive of autonomic dysfunction. In one study, 16 patients were assessed for dysfunction using the SCOPA-AUT scale. They found that all five subscales were affected in PSP, with significant differences in urinary control and storage compared to PD and MSA patients (Wang et al., 2025). Evidence from other studies have reported bladder dysfunction, dysphagia, and constipation, and additionally, tests confirmed sudomotor and pupillomotor abnormalities, which implies damage to small autonomic fibers (Baschieri et al., 2023). When it comes to small fibers that innervate the skin, significant length-dependent loss of autonomic fibers compared to controls has been found in PSP patients and correlates with functional impairment and disease severity (Dubbioso et al., 2021).

#### Peripheral tau protein in PSP

Elevations in hyperphosphorylated tau are believed to be the causative agent in PSP. Tauopathies, such as PSP, feature abnormal tau protein deposits in the CNS and PNS, including DRG (Vacchi et al., 2022; Dugger et al., 2019; Mellone et al., 2013) (Table 5). In an autopsy study of PSP patients, 60% had tau pathology in their posterior spinal roots and surrounding peripheral nerves. In the PNS, tau is more prevalent and prominent in PSP compared to diseases like AD. PSP patients had PNS-tau lesions that appeared as large fibrils similar to NFTs in the brain (Tanaka et al., 2024). Also, tau is elevated in the CSF of patients with PSP compared with healthy controls (Arai et al., 2004). Small-fiber neuropathy in PSP may be related to pathological tau in peripheral nerves.

#### Summary

PSP is a rare, rapidly progressive 4R tauopathy characterized by early axial rigidity, falls, cognitive decline, and a range of motor and non-motor symptoms. The disease is driven by abnormal tau accumulation in the CNS, but potentially also the PNS. Increasing evidence highlights significant peripheral involvement, including sensory disturbances, autonomic dysfunction, and pain, with up to 80% of patients exhibiting sensory dysfunction, 11%−68% exhibiting pain, and up to 65.2% of patients exhibiting small-fiber neuropathy, both autonomic and sensory, according to discussed studies. Structural and functional studies demonstrate widespread peripheral nerve degeneration and inflammation, suggesting that PSP is not solely a central neurodegenerative disorder but also involves substantial PNS pathology. Further exploration and therapeutic interventions targeting the PNS may prove beneficial in slowing disease progression.

**Table 5:** Summary of sensory dysfunction, pain, and small-fiber neuropathy in PSP.

Progressive supranuclear palsy	
Primary pathologic protein (s)	Tau
Prevalence of sensory disturbances	Up to 80%
Prevalence of pain	11%−68%
Prevalence of small-fiber neuropathy	Up to 65%

Prevalence of disturbances (such as loss of smell, loss of hearing, loss of feeling to touch), pain, and small-fiber peripheral neuropathy. Sensory abnormalities and small-fiber degeneration may be linked to the pathologic proteins underlying PSP. Prevalences are based on findings from several publications discussed in the text.

## Frontotemporal dementia

### Disease overview

Frontotemporal lobar degeneration (FTLD) is characterized by pathological changes in the frontal and temporal lobes. Frontotemporal dementia (FTD), also known as Pick's Disease, is a progressive neurodegenerative disease that also features degeneration in these regions and is the third most common dementia. FTD includes abnormalities in behavior, executive function, and language (Bang et al., 2015). A recent systematic search of population-based studies estimating prevalence or incidence of FTD resulted in 32 eligible studies with a pooled incidence of 2.28 per 100,000 person-years and prevalence (Urso et al., 2025). FTD includes several disease subtypes, including a behavioral variant (bvFTD), primary aphasia, right lobe-focused, and motor neuron-associated forms (Antonioni et al., 2023; Ghosh and Lippa, 2015). Additionally, there are overlaps of motor neuron disease and FTD seen often clinically (Devenney et al., 2015). Both TDP-43 and tau are known to accumulate and contribute to pathology in FTD, with recent focus on plasma extracellular vesicles containing these proteins as diagnostic biomarkers (Chatterjee et al., 2024) (Table 2). Currently, there is no cure for FTD and no significant treatments to slow down progression. Symptoms can be managed through SSRIs and speech therapy (Tsai and Boxer, 2014). It is important to understand the complex neuropathological heterogeneity and presentations of FTD and bring more attention to underappreciated complications such as peripheral nerve degeneration, sensory abnormalities, and abnormal proteins throughout the whole body.

### Abnormalities in sensation in FTD

Patients with FTD typically have their physical senses intact and suffer more from social and emotional processing (Marshall et al., 2019). However, pain is widely prevalent. According to one longitudinal study of 97 FTD patients, it was found that 40.2% have either somatic complaints and/or an altered response to pain (Landqvist Waldö et al., 2014) (Table 6). A separate cross-sectional analysis found that six out of 11 FTD subjects tested reported pain, with the majority reporting mild levels (Atee et al., 2021) (Table 6). Common incidences of somatic complaints such as musculoskeletal, abdominal, and headache pain (Landqvist Waldö et al., 2014) (Figure 1) is likely due in some part to central processing, but sensory nociceptors are nevertheless affected as well in FTD. Nociceptive sensitivity changes can range from hypersensitivity to hyposensitivity, with some patients exhibiting allodynia and others hypoalgesia or an increased pain threshold (Carlino et al., 2010) (Figure 1). bvFTD patients more commonly exhibit decreased responses to temperature (Fletcher et al., 2015). This could be due to systemic proinflammatory activation in FTD. For example, high levels of interleukins, including IL-2, and factors in the tumor necrosis factor superfamily were found in the plasma of bvFTD patients (Chu et al., 2024). However, there is also pre-clinical evidence of small-fiber degeneration and proteinopathy in peripheral nerve tissue.

## Small-fiber neuropathy and peripheral tau protein in FTD

There is little clinical evidence of small-fiber neuropathy in FTD patients. To our knowledge, only one study has explored this, which looked at corneal nerve fibers. This group found that corneal nerve occupancy was reduced in the stroma of FTD patients; however, there were only slight reductions on average in sub-basal plexus corneal nerve fiber length compared to control subjects (Table 6). On the preclinical side, they found that hTau mice had both increased phosphorylated tau in the sciatic nerve and significantly reduced IENFD and stromal corneal nerve length (Marquez et al., 2021) (Table 6). While pTau was found to be increased in peripheral nerve preclinically, there is not much evidence clinically. It is known, however, that plasma tau is increased (Foiani et al., 2018). Further studies need to be done to determine if small-fiber neuropathy is a common occurrence, and whether this relates to abnormal protein buildup in peripheral tissue.

### Summary

These phenotypes in the PNS of patients with FTD highlight a frequently understudied component of the disease, with limited understanding of both the mechanisms of degeneration and their contribution to the overall disease. FTD is a dementia-provoking disease characterized by the accumulation of both misfolded TDP-43 and tau protein, with increased pTau found in peripheral tissues. Sensory disturbances and pain are reported in 40.2%−54% of FTD subjects according to the discussed clinical studies. Small-fiber neuropathy is less commonly identified, but there is evidence of corneal fiber degeneration clinically and IENF degeneration preclinically. Further exploration and therapeutic interventions targeting the PNS may prove beneficial in slowing disease progression.

**Table 6:** Summary of sensory dysfunction, pain, and small-fiber neuropathy in FTD.

Frontotemporal dementia	
Primary pathologic protein (s)	TDP-43, Tau
Prevalence of sensory disturbances	Up to 40%
Prevalence of pain	54%−71%
Prevalence of small-fiber neuropathy	Reduced corneal nerve fiber occupancy

Prevalence of disturbances (such as loss of smell, loss of hearing, loss of feeling to touch), pain, and small-fiber peripheral neuropathy. Sensory abnormalities and small-fiber degeneration may be linked to the pathologic proteins underlying FTD. Prevalences are based on findings from several publications discussed in the text.

## Corticobasal degeneration

### Disease overview

Corticobasal degeneration (CBD) is another rare neurodegenerative disease that features defects in movement, speech, memory, and swallowing (Armstrong et al., 2013). The classic clinical presentation, corticobasal syndrome (CBS), presents as rigidity and dystonia along with the common alien limb phenomenon and is typically idiopathic (Grijalvo-Perez and Litvan, 2014). Pooled evidence from nine studies in CBS demonstrates a prevalence of 2.3 per 100,000 people per year (Swallow et al., 2022). CBD is a primary 4R tauopathy (Kouri et al., 2011) (Table 7), with some distinct cases of TDP-43 build-up as well (Koga et al., 2018). In a study with 19 patients with CBS and 19 matched controls, it was clear that both decreased interconnectedness in brain regions such as the middle temporal gyrus and posterior insula and increases in connectivity in the anterior cingulum, medial superior- frontal gyrus, and bilateral caudate nuclei distinguished CBS patients from controls. Structural abnormalities such as reduced gray matter volume in the thalamus, bilateral insula, and putamen also outlined biomarkers of CBS (Ballarini et al., 2020). Therapeutic candidates targeting tau pathology are currently being studied in clinical trials (Nouh and Younes, 2024), however, there is no current cure. Treatments such as muscle relaxers and botox can be used for the alleviation of motor defects, but the complex neuropathological heterogeneity of CBD requires more understanding of how the disease progresses systemically, such as greater attention toward comorbidities such as sensory dysfunction.

### Abnormalities in sensation and peripheral tau protein in CBD

Around 25%−33% of cases involve cortical sensory loss (Armstrong et al., 2013; Kompoliti et al., 1998) (Table 7). Persistent pain seems to be common in CBD, with around 33.3% of study participants of a total of 27 tested (Ohtomo et al., 2016) (Table 3). Pain is often reported as dystonia-related, but neuropathic pain is also seen (Ozsancak et al., 2000; Unti et al., 2019) (Figure 1). Additionally, persistent visceral pain has been observed (281) (Figure 1). One study showed that about 20.8% of patients with CBD had evidence of peripheral triggers that preceded symptom onset (Lenka and Jankovic, 2025). While incidences of small-fiber neuropathy are unclear in CBD, nociception could imply underlying subclinical changes in peripheral nerves, too. There is some evidence of changes in thermal sensation seen (Koga et al., 2022) (Figure 1). CBD is similar to PSP, although there are differences in tau proteolysis, and this could play a role in variability in neuropathy incidence and severity (Arai et al., 2004). Evidence of peripheral tau protein being highly expressed in both the ankle and cervical skin sites of CBD (Dellarole et al., 2024) (Table 7) raises question of whether proteinopathy occurs in the PNS too, and in theory, can also affect peripheral nerve, although many more studies are needed to support this idea.

### Summary

These phenotypes in the PNS of patients with CBD highlight a frequently understudied component of the disease, with limited understanding of both the mechanisms of degeneration and their contribution to the overall disease. Cortical sensory loss is common, and pain is reported in up to 33% of people with CBD according to one study. There is no evidence to our knowledge of small-fiber neuropathy in CBD. Further exploration and therapeutic interventions targeting the PNS and the tau protein in peripheral tissues may prove beneficial in slowing disease progression.

**Table 7:** Summary of sensory dysfunction, pain, and small-fiber neuropathy in CBD.

Corticobasal degeneration	
Primary pathologic protein (s)	Tau
Prevalence of sensory disturbances	25%−33%
Prevalence of pain	33.30%
Prevalence of small-fiber neuropathy	Unclear

Prevalence of disturbances (such as loss of smell, loss of hearing, loss of feeling to touch), pain, and small-fiber peripheral neuropathy. Sensory abnormalities and small-fiber degeneration may be linked to the pathologic proteins underlying CBD. Prevalences are based on findings from several publications discussed in the text.

## Huntington's disease

### Disease overview

Huntington's disease (HD) is an inherited neurodegenerative disorder that leads to progressive neuronal wasting and affects around 41,000 people in the US, according to Dr. Lisa Toran at Confluence Health. A recent meta-analysis showed pooled evidence of 0.48 cases per 100,000 person-years globally (Medina et al., 2022). The HD gene was identified in 1993 (The Huntington's Disease Collaborative Research Group, 1993), and this gene of single origin has had detrimental effects. The Huntingtin protein (htt) is essential in this disease, and abnormal misfolding contributes to pathology (Schulte and Littleton, 2011). Neuronal loss in the striatum and cortex, typical regions affected, primarily characterizes HD (Vonsattel et al., 1985), although other regions are affected too. Common symptoms of HD include cognitive dysfunction, depression, and chorea, all of which often precede motor dysfunction (Paulsen et al., 2008). While there is no cure for HD, treatments such as VMAT2 inhibitors or antipsychotic drugs aim to help with the motor and movement symptoms (Frank, 2014). It is important to understand this disease and outline all observed complications in more detail on a systemic level.

### Abnormalities in sensation in HD

In a study of 44 patients at risk for HD as well as 17 already diagnosed, it was found that early sensory-perceptual changes are a strong predictor of disease, with measurable changes in sensory function resulting in a 95.1% correct classification rate of those at risk (Hayward et al., 1985). A prominent complication in HD is dysfunction in olfactory recognition early in the disease, pointing to early sensory abnormalities (Bacon Moore et al., 1999; Moberg et al., 1987). Olfaction seems not to be the only sensory modality affected, as pain is seemingly an underestimated problem (Scherder and Statema, 2010; Andrich et al., 2009). A study of 33 surveys completed by juvenile HD patients showed that 60% reported somatosensory defects (itching), and 69% reported pain (Moser et al., 2017) (Table 8). In a data mining study, overall pain prevalence for carriers of the HD gene was found to be 41% of the 1474 people from whom data was collected (Underwood et al., 2017). There are reports of abdominal, headache, back, and limb pain in HD, but also neuropathic tactile allodynia (Li et al., 2023) (Figure 1). While pain could be due to abnormal sensory processing that is known to be altered in HD (Boecker et al., 1999; Sprenger et al., 2026), there are also cases of nociception. One report highlighted two HD cases in which one patient suffered from sensory “bee-sting pain” and the other complained of sharp, tingling pain in their legs and arms (Albin and Young, 1988). Additionally, multiple studies have shown dysfunction of nociceptive signal transmission at the spinal cord level in mid-stage HD, suggesting that subclinical sensory changes are present (Perrotta et al., 2012; De Tommaso et al., 2011) (Figure 1).

### Peripheral huntingtin protein in HD

The mutated HTT gene is the main cause of HD, and intracellular protein aggregates in HTT-mutated cells are present in the PNS (Sassone et al., 2009; Marques Sousa and Humbert, 2013). As discussed in another review, also highlighting abnormal htt in peripheral tissue, it is plausible that peripheral neurons can uptake this protein and peripheral to central transmission may occur (Chuang and Demontis, 2021) (Table 8). Additionally, HAP1, a neuronal scaffolding protein that features enhanced binding to htt, is also enriched in DRG (Islam et al., 2020) and has also interestingly been found to modulate nociception pre-clinically (Gloor et al., 2022). The presence of htt in peripheral tissue, the role of HAP1 in both abnormal htt and nociceptive reception which is seen in HD, and abnormal glucose homeostasis in the peripheral tissues of patients with HD (Sassone et al., 2009) suggest possible underlying peripheral neuropathy in some HD patients with small fibers likely involved.

### Summary

These phenotypes in the PNS of patients with HD highlight a frequently understudied component of the disease, with limited understanding of both the mechanisms of degeneration and their contribution to the overall disease. Sensory disturbances may be an early predictor of disease, and according to one study discussed, pain affects up to 69% of people with HD. Although the universal prevalence of small-fiber neuropathy in HD is unclear, peripheral tissues are pathologically affected. Further exploration and therapeutic interventions targeting the PNS may prove beneficial in slowing disease progression.

**Table 8:** Summary of sensory dysfunction, pain, and small-fiber neuropathy in HD.

Huntington's disease	
Primary pathologic protein (s)	Huntingtin
Prevalence of sensory disturbances	Up to 60%
Prevalence of pain	Up to 69%
Prevalence of small-fiber neuropathy	Abnormalities in peripheral tissue are common

Prevalence of disturbances (such as loss of smell, loss of hearing, loss of feeling to touch), pain, and small-fiber peripheral neuropathy. Sensory abnormalities and small-fiber degeneration may be linked to the pathologic proteins underlying HD. Prevalences are based on findings from several publications discussed in the text.

**Figure 1:**
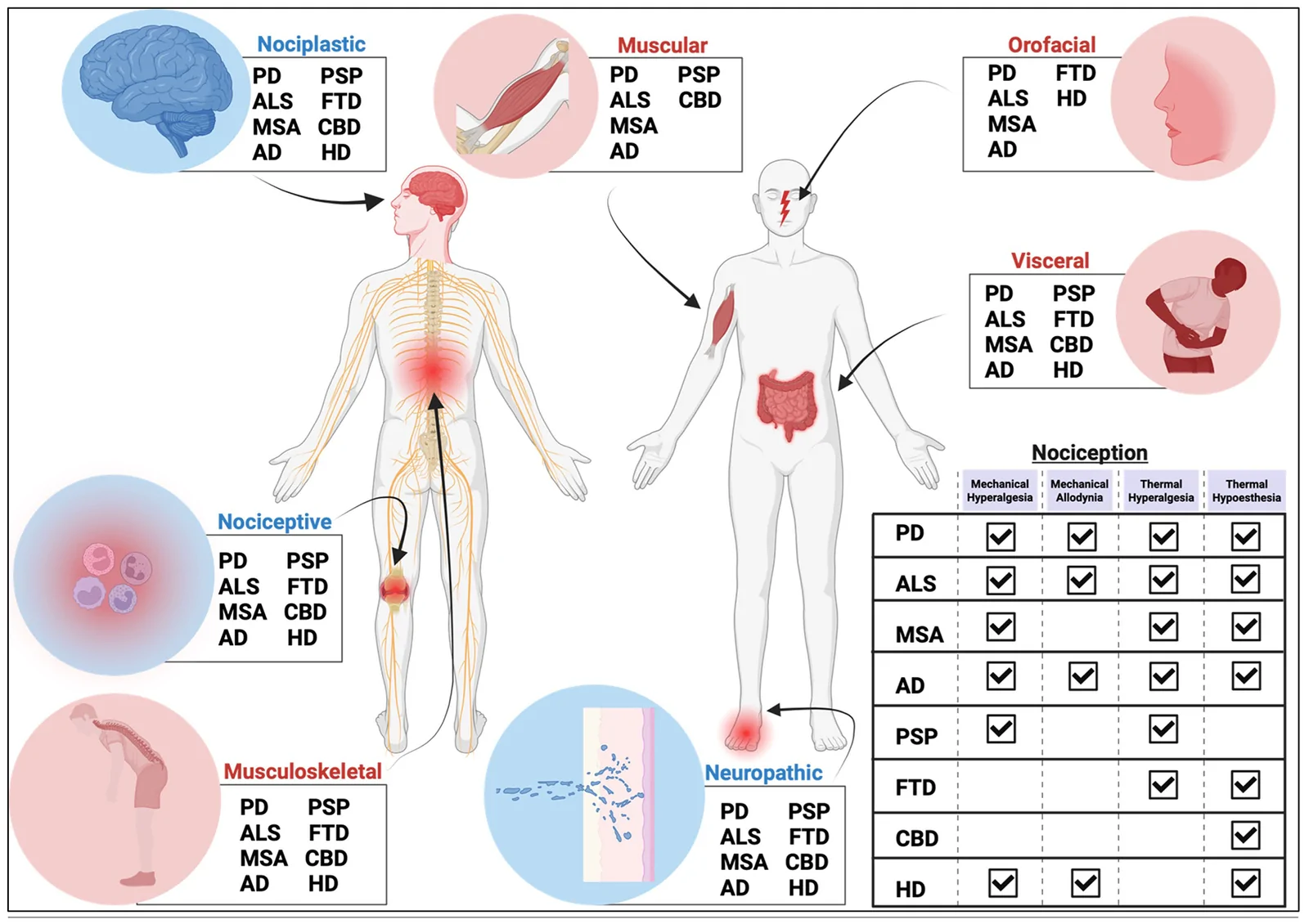
Pain phenotypes across the discussed diseases. The three principal categories of pain (nociplastic, neuropathic, and nociceptive) are highlighted in blue. Also included is a table of specific sensory states related to nociception across diseases, identified from publications discussed in the text. Additionally, other underreported pain phenotypes (muscular, orofacial, visceral, musculoskeletal) are highlighted in red. Created in BioRender. Wright, D. (2026) https://BioRender.com/90bjde4. Licensed under Individual – Academic RR2A7DBQGK. Source: BioRender.

### Conclusion

The purpose of this review is to highlight sensory involvement across progressive central neurodegenerative diseases, including pain, sensory dysfunction, and neuropathy. It is undoubtedly true that pain and sensory dysfunction are subclinical complications in a wide variety of pathologies, as aging brings forth a long list of comorbidities. However, to understand why some people undergo more degeneration and decline, both centrally and peripherally, than others, it is crucial to appreciate commonalities seen across individuals of these diseases to further distinguish etiology. So, it is important to discuss that comorbidities such as pain are prevalent across these diseases, and while it is often not focused on, it plausibly can worsen disease progression. Small-fiber peripheral neuropathy, both sensory and autonomic, is also an underappreciated manifestation across these diseases, with peripheral nerve degeneration likely playing a role in nociception. Disruption of peripheral sensory neurons and their metabolic and inflammatory environments may contribute to disease susceptibility and progression, warranting further investigation. These peripheral nerve phenotypes represent an underrecognized component of disease, with limited understanding of their mechanisms and contributions to progression, but they may offer novel therapeutic targets. Plasma- and CSF-based biomarkers of pathogenic proteins are emerging as key diagnostic tools, and peripheral sources—including sensory neurons and skin biopsies—may provide additional, less invasive insights into disease-specific pathology.

Beyond biomarkers, sensory systems such as olfactory, visual, auditory, and enteric pathways are often disrupted early in disease, with growing evidence implicating somatic sensory neurons and DRG in disease-related dysfunction. Peripheral sensitization, driven by protein accumulation (e.g., Aβ, tau, α-synuclein, TDP-43), inflammation, and metabolic stress, likely alters neuronal signaling and contributes to both pain and neurodegeneration. DRG neurons interact closely with the immune system and participate in threat surveillance, suggesting that persistent sensory dysfunction may influence central processing through bottom-up signaling mechanisms. Ultimately, a deeper understanding of sensory small-fiber involvement could be essential for understanding these diseases more systemically. Additionally, further study may help to improve diagnosis, identify therapeutic targets, and enhance quality of life for affected individuals.

## References

[B1] Abele M. Schulz J. B. Bürk K. Topka H. Dichgans J. Klockgether T. (2000). Nerve conduction studies in multiple system atrophy. Eur. Neurol. 43, 221–223. doi: 10.1159/000008179 10828652

[B2] Abraham A. Barnett C. Katzberg H. D. Lovblom L. E. Perkins B. A. Bril V. (2018). Sex differences in neuropathic pain intensity in diabetes. J. Neurol. Sci. 388, 103–106. doi: 10.1016/j.jns.2018.03.008 29627001

[B3] Abu-Rumeileh S. Scholle L. Mensch A. Großkopf H. Ratti A. Kölsch A. . (2025). Phosphorylated tau 181 and 217 are elevated in serum and muscle of patients with amyotrophic lateral sclerosis. Nat. Commun. 16:2019. doi: 10.1038/s41467-025-57144-7 40044663 PMC11882981

[B506] Achterberg W. P. Pieper M. J. van Dalen-Kok A. H. de Waal M. W. Husebo B. S. Lautenbacher S. . (2013). Pain management in patients with dementia. Clin. Interv. Aging. 8, 1471–1482. doi: 10.2147/CIA.S36739 PMC3817007 24204133

[B4] Agah E. Mojtabavi H. Behkar A. Heidari A. Ajdari A. Shaka Z. . (2024). CSF and blood levels of neurofilaments, T-Tau, P-Tau, and Abeta-42 in amyotrophic lateral sclerosis: a systematic review and meta-analysis. J. Transl. Med. 22:953. doi: 10.1186/s12967-024-05767-7 39434139 PMC11492992

[B5] Agarwal S. Gilbert R. (2025). Progressive Supranuclear Palsy. StatPearls. Treasure Island, FL: StatPearls Publishing. 30252354

[B6] Aisen P. S. Davis K. L. (1994). Inflammatory mechanisms in Alzheimer's disease: implications for therapy. Am. J. Psychiatry 151, 1105–1113. doi: 10.1176/ajp.151.8.1105 7518651

[B7] Albers M. W. Gilmore G. C. Kaye J. Murphy C. Wingfield A. Bennett D. A. . (2015). At the interface of sensory and motor dysfunctions and Alzheimer's disease. Alzheimers Dement. 11, 70–98. doi: 10.1016/j.jalz.2014.04.514 25022540 PMC4287457

[B8] Albin R. L. Young A. B. (1988). Somatosensory phenomena in Huntington's disease. Mov Disord. 3, 343–346. doi: 10.1002/mds.870030411 2974928

[B9] Ali M. Timsina J. Western D. Liu M. Beric A. Budde J. . (2025). Multi-cohort cerebrospinal fluid proteomics identifies robust molecular signatures across the Alzheimer disease continuum. Neuron 113, 1363–1379.e9. doi: 10.1016/j.neuron.2025.02.014 40088886 PMC12067247

[B10] Alkandari M. Hollywood A. (2023). People's experiences living with peripheral neuropathy: a qualitative study. Front. Pain Res. 4:1162405. doi: 10.3389/fpain.2023.1162405 37449296 PMC10338106

[B11] Andrade-Guerrero J. Santiago-Balmaseda A. Jeronimo-Aguilar P. Vargas-Rodríguez I. Cadena-Suárez A. R. Sánchez-Garibay C. . (2023). Alzheimer's disease: an updated overview of its genetics. Int. J. Mol. Sci. 24:3754. doi: 10.3390/ijms24043754 36835161 PMC9966419

[B12] Andréasson M. Paslawski W. Terkelsen A. J. Samuelsson K. Zetterberg H. Blennow K. . (2025). Cutaneous nerve fiber pathology and function in Parkinson's disease and atypical parkinsonism - a cohort study. NPJ Parkinsons Dis. 11:170. doi: 10.1038/s41531-025-01030-y 40517154 PMC12167382

[B13] Andrich J. E. Wobben M. Klotz P. Goetze O. Saft C. (2009). Upper gastrointestinal findings in Huntington's disease: patients suffer but do not complain. J. Neural Transm. 116, 1607–1611. doi: 10.1007/s00702-009-0310-1 19771391

[B14] Ansari U. Alam M. Nadora D. Muttalib Z. Chen V. Taguinod I. . (2024). Assessing the efficacy of amyotrophic lateral sclerosis drugs in slowing disease progression: a literature review. AIMS Neurosci. 11, 166–177. doi: 10.3934/Neuroscience.2024010 38988889 PMC11230861

[B15] Antonioni A. Raho E. M. Lopriore P. Pace A. P. Latino R. R. Assogna M. . (2023). Frontotemporal dementia, where do we stand? A narrative review. Int. J. Mol. Sci. 24:11732. doi: 10.3390/ijms241411732 37511491 PMC10380352

[B16] Arai T. Ikeda K. Akiyama H. Nonaka T. Hasegawa M. Ishiguro K. . (2004). Identification of amino-terminally cleaved tau fragments that distinguish progressive supranuclear palsy from corticobasal degeneration. Ann. Neurol. 55, 72–79. doi: 10.1002/ana.10793 14705114

[B17] Arena J. E. Weigand S. D. Whitwell J. L. Hassan A. Eggers S. D. Höglinger G. U. . (2016). Progressive supranuclear palsy: progression and survival. J. Neurol. 263, 380–389. doi: 10.1007/s00415-015-7990-2 26705121

[B18] Armstrong M. J. Litvan I. Lang A. E. Bak T. H. Bhatia K. P. Borroni B. . (2013). Criteria for the diagnosis of corticobasal degeneration. Neurology 80, 496–503. doi: 10.1212/WNL.0b013e31827f0fd1 23359374 PMC3590050

[B19] Atee M. Morris T. Macfarlane S. Cunningham C. (2021). Pain in dementia: prevalence and association with neuropsychiatric behaviors. J. Pain Symptom Manage. 61, 1215–1226. doi: 10.1016/j.jpainsymman.2020.10.011 33068708

[B20] Atmaca A. Ketenci A. Sahin I. Sengun I. S. Oner R. I. Erdem Tilki H. . (2024). Expert opinion on screening, diagnosis and management of diabetic peripheral neuropathy: a multidisciplinary approach. Front. Endocrinol. 15:1380929. doi: 10.3389/fendo.2024.1380929 38952393 PMC11215140

[B21] Awal G. Kaur S. (2018). Association of cutaneous amyloidosis with neurodegenerative amyloidosis: correlation or coincidence? J. Clin. Aesthet. Dermatol. 11, 25–27. PMC5891083 29657668

[B22] Ayers J. I. Cashman N. R. (2018). Prion-like mechanisms in amyotrophic lateral sclerosis. Handb. Clin. Neurol. 153, 337–354. doi: 10.1016/B978-0-444-63945-5.00018-0 29887144

[B23] Azhary H. Farooq M. U. Bhanushali M. Majid A. Kassab M. Y. (2010). Peripheral neuropathy: differential diagnosis and management. Am. Fam. Physician. 81, 887–892. 20353146

[B24] Bacon Moore A. S. Paulsen J. S. Murphy C. (1999). A test of odor fluency in patients with Alzheimer's and Huntington's disease. J. Clin. Exp. Neuropsychol. 21, 341–351. doi: 10.1076/jcen.21.3.341.918 10474173

[B25] Ballarini T. Albrecht F. Mueller K. Jech R. Diehl-Schmid J. Fliessbach K. . (2020). Disentangling brain functional network remodeling in corticobasal syndrome - A multimodal MRI study. Neuroimage Clin. 25:102112. doi: 10.1016/j.nicl.2019.102112 31821953 PMC6906725

[B26] Bang J. Spina S. Miller B. L. (2015). Frontotemporal dementia. Lancet 386, 1672–1682. doi: 10.1016/S0140-6736(15)00461-4 26595641 PMC5970949

[B27] Barboza V. R. Kubota G. T. da Silva V. A. Barbosa L. M. Arnaut D. Rodrigues A. L. L. . (2023). Parkinson's disease-related pains are not equal: clinical, somatosensory and cortical excitability findings in individuals with nociceptive pain. J. Pain 24, 2186–2198. doi: 10.1016/j.jpain.2023.07.005 37442404

[B603] Barclay C. L. Lang A. E. (1997). Dystonia in progressive supranuclear palsy. J. Neurol. Neurosurg. Psychiatry 62, 352–356. doi: 10.1136/jnnp.62.4.352 PMC1074090 9120447

[B28] Barragán-Iglesias P. Kuhn J. Vidal-Cantú G. C. Salinas-Abarca A. B. Granados-Soto V. Dussor G. O. . (2019). Activation of the integrated stress response in nociceptors drives methylglyoxal-induced pain. Pain 160, 160–171. doi: 10.1097/j.pain.0000000000001387 30157134 PMC6800106

[B29] Baschieri F. Vitiello M. Cortelli P. Calandra-Buonaura G. Morgante F. (2023). Autonomic dysfunction in progressive supranuclear palsy. J Neurol. 270, 109–129. doi: 10.1007/s00415-022-11347-w 36042018 PMC9813233

[B30] Beiske A. G. Loge J. H. Rønningen A. Svensson E. (2009). Pain in Parkinson's disease: prevalence and characteristics. Pain 141, 173–177. doi: 10.1016/j.pain.2008.12.004 19100686

[B31] Bell T. R. Franz C. E. Eyler L. T. Fennema-Notestine C. Puckett O. K. Dorros S. M. . (2024). Probable chronic pain, brain structure, and Alzheimer's plasma biomarkers in older men. J. Pain 25:104463. doi: 10.1016/j.jpain.2024.01.006 38199594 PMC11740721

[B32] Bloom A. J. Mao X. Strickland A. Sasaki Y. Milbrandt J. DiAntonio A. (2022). Constitutively active SARM1 variants that induce neuropathy are enriched in ALS patients. Mol. Neurodegener. 17:1. doi: 10.1186/s13024-021-00511-x 34991663 PMC8739729

[B33] Boecker H. Ceballos-Baumann A. Bartenstein P. Weindl A. Siebner H. R. Fassbender T. . (1999). Sensory processing in Parkinson's and Huntington's disease: investigations with 3D HO-PET. Brain. 122 (Pt 9), 1651–1665. doi: 10.1093/brain/122.9.1651 10468505

[B502] Boesch S. M. Wenning G. K. Ransmayr G. Poewe W. (2002). Dystonia in multiple system atrophy. J. Neurol. Neurosurg. Psychiatry 72, 300–303. doi: 10.1136/jnnp.72.3.300 PMC1737793 11861684

[B34] Bombaci A. Lupica A. Pozzi F. E. Remoli G. Manera U. Di Stefano V. . (2023). Sensory neuropathy in amyotrophic lateral sclerosis: a systematic review. J. Neurol. 270, 5677–5691. doi: 10.1007/s00415-023-11954-1 37610446 PMC10632209

[B35] Bornier N. Mulliez A. Chenaf C. Elyn A. Teixeira S. Authier N. . (2023). Chronic pain is a risk factor for incident Alzheimer's disease: a nationwide propensity-matched cohort using administrative data. Front. Aging Neurosci. 15:1193108. doi: 10.3389/fnagi.2023.1193108 37842123 PMC10575742

[B36] Bradford D. Rodgers K. E. (2024). Advancements and challenges in amyotrophic lateral sclerosis. Front. Neurosci. 18:1401706. doi: 10.3389/fnins.2024.1401706 38846716 PMC11155303

[B37] Brenowitz W. D. Kaup A. R. Lin F. R. Yaffe K. (2019). Multiple sensory impairment is associated with increased risk of dementia among black and white older adults. J. Gerontol. A Biol. Sci. Med. Sci. 74, 890–896. doi: 10.1093/gerona/gly264 30452551 PMC6521912

[B38] Buchholz E. Machule M. L. Buthut M. Stefanovski L. Rössling R. Prüss H. (2025). Overlapping presence of β-amyloid, tau, p-tau, and α-synuclein in skin nerve fibers in Alzheimer's disease. J. Neurol. 272:247. doi: 10.1007/s00415-025-12994-5 40042672 PMC11882635

[B39] Cacace R. Sleegers K. Van Broeckhoven C. (2016). Molecular genetics of early-onset Alzheimer's disease revisited. Alzheimers Dement. 12, 733–748. doi: 10.1016/j.jalz.2016.01.012 27016693

[B40] Callaghan B. C. Reynolds E. Banerjee M. Chant E. Villegas-Umana E. Feldman E. L. (2020). Central obesity is associated with neuropathy in the severely obese. Mayo Clin. Proc. 95, 1342–1353. doi: 10.1016/j.mayocp.2020.03.025 32622444 PMC7340115

[B500] Campese N. Caliò B. Leys F. Kaltenbach L. Göbel G. Wanschitz J. . (2023). Pain in multiple system atrophy a systematic review and meta-analysis. Mov. Disord. Clin. Pract. 10, 1738–1749. doi: 10.1002/mdc3.13897 PMC10715371 38094640

[B41] Campese N. Göbel G. Wanschitz J. Schlager A. Caliò B. Leys F. . (2024). Pain in multiple system atrophy: a community-based survey. Mov. Disord. 39, 1784–1798. doi: 10.1002/mds.29961 39101334

[B42] Cao S. Fisher D. W. Yu T. Dong H. (2019). The link between chronic pain and Alzheimer's disease. J. Neuroinflammation 16:204. doi: 10.1186/s12974-019-1608-z 31694670 PMC6836339

[B43] Carlino E. Benedetti F. Rainero I. Asteggiano G. Cappa G. Tarenzi L. . (2010). Pain perception and tolerance in patients with frontotemporal dementia. Pain 151, 783–789. doi: 10.1016/j.pain.2010.09.013 20934811

[B44] Castelli G. Desai K. M. Cantone R. E. (2020). Peripheral neuropathy: evaluation and differential diagnosis. Am. Fam. Physician. 102, 732–739. 33320513

[B45] Catumbela C. S. G. Giridharan V. V. Barichello T. Morales R. (2023). Clinical evidence of human pathogens implicated in Alzheimer's disease pathology and the therapeutic efficacy of antimicrobials: an overview. Transl. Neurodegener. 12:37. doi: 10.1186/s40035-023-00369-7 37496074 PMC10369764

[B46] Chaithra S. P. Prasad S. Holla V. V. Stezin A. Kamble N. Yadav R. . (2020). The non-motor symptom profile of progressive supranuclear palsy. J. Mov. Disord. 13, 118–126. doi: 10.14802/jmd.19066 32241079 PMC7280946

[B47] Chatterjee M. Özdemir S. Fritz C. Möbius W. Kleineidam L. Mandelkow E. . (2024). Plasma extracellular vesicle tau and TDP-43 as diagnostic biomarkers in FTD and ALS. Nat. Med. 30, 1771–1783. doi: 10.1038/s41591-024-02937-4 38890531 PMC11186765

[B48] Chiò A. Mora G. Lauria G. (2017). Pain in amyotrophic lateral sclerosis. Lancet Neurol. 16, 144–157. doi: 10.1016/S1474-4422(16)30358-1 27964824

[B49] Chipika R. H. Mulkerrin G. Murad A. Lope J. Hardiman O. Bede P. (2022). Alterations in somatosensory, visual and auditory pathways in amyotrophic lateral sclerosis: an under-recognised facet of ALS. J. Integr. Neurosci. 21:88. doi: 10.31083/j.jin2103088 35633169

[B50] Chowdhury A. Mukherjee A. Sinharoy U. Pandit A. Biswas A. (2021). Non-motor features of amyotrophic lateral sclerosis: a clinic-based study. Ann. Indian Acad. Neurol. 24, 745–753. doi: 10.4103/aian.AIAN_51_21 35002134 PMC8680868

[B51] Chu M. Jiang D. Li D. Yan S. Liu L. Nan H. . (2024). Atrophy network mapping of clinical subtypes and main symptoms in frontotemporal dementia. Brain 147, 3048–3058. doi: 10.1093/brain/awae067 38426222 PMC11370799

[B52] Chuang C. L. Demontis F. (2021). Systemic manifestation and contribution of peripheral tissues to Huntington's disease pathogenesis. Ageing Res. Rev. 69:101358. doi: 10.1016/j.arr.2021.101358 33979693 PMC8205985

[B53] Clos A. L. Kayed R. Lasagna-Reeves C. A. (2012). Association of skin with the pathogenesis and treatment of neurodegenerative amyloidosis. Front. Neurol. 3:5. doi: 10.3389/fneur.2012.00005 22319507 PMC3262151

[B54] Colosimo C. Morgante L. Antonini A. Barone P. Avarello T. P. Bottacchi E. . (2010). Non-motor symptoms in atypical and secondary parkinsonism: the PRIAMO study. J. Neurol. 257, 5–14. doi: 10.1007/s00415-009-5255-7 19669613

[B55] Corrà M. F. Vila-Chã N. Sardoeira A. Hansen C. Sousa A. P. Reis I. . (2023). Peripheral neuropathy in Parkinson's disease: prevalence and functional impact on gait and balance. Brain 146, 225–236. doi: 10.1093/brain/awac026 35088837 PMC9825570

[B56] Cortés N. Andrade V. Guzmán-Martínez L. Estrella M. Maccioni R. B. (2018). Neuroimmune tau mechanisms: their role in the progression of neuronal degeneration. Int. J. Mol. Sci. 19:956. doi: 10.3390/ijms19040956 29570615 PMC5979395

[B600] Cui Z. Guo Z. Wei L. Zou X. Zhu Z. Liu Y. . (2022). Altered pain sensitivity in 5 × familial Alzheimer disease mice is associated with dendritic spine loss in anterior cingulate cortex pyramidal neurons. Pain 163, 2138–2153. doi: 10.1097/j.pain.0000000000002648 PMC9578529 35384934

[B57] Dalla Bella E. Lombardi R. Porretta-Serapiglia C. Ciano C. Gellera C. Pensato V. . (2016). Amyotrophic lateral sclerosis causes small fiber pathology. Eur. J. Neurol. 23, 416–420. doi: 10.1111/ene.12936 26806218

[B58] Daroff R. B. (1987). Progressive supranuclear palsy: a brief personalized history. Yale J Biol Med. 60, 119–122. 3577208 PMC2590314

[B59] de Araújo D. F. de Melo Neto A. P. Oliveira Í. S. Brito B. S. de Araújo I. T. Barros I. S. . (2016). Small (autonomic) and large fiber neuropathy in Parkinson disease and parkinsonism. BMC Neurol. 16:139. doi: 10.1186/s12883-016-0667-3 27530902 PMC4988006

[B504] de Siqueira S. Rolim T. S. Teixeira M. J. Nitrini R. Anghinah R. de Siqueira J. T. T. (2010). Oral infections and orofacial pain in Alzheimer's disease: case report and review. Dement. Neuropsychol. 4, 145–150. doi: 10.1590/S1980-57642010DN40200012 PMC5619174 29213678

[B60] De Tommaso M. Serpino C. Difruscolo O. Cormio C. Sciruicchio V. Franco G. . (2011). Nociceptive inputs transmission in Huntington's disease: a study by laser evoked potentials. Acta Neurol. Belg. 111, 33–40. 21510231

[B61] Debnath M. Dey S. Sreenivas N. Pal P. K. Yadav R. (2022). Genetic and epigenetic constructs of progressive supranuclear palsy. Ann. Neurosci. 29, 177–188. doi: 10.1177/09727531221089396 36419517 PMC9676335

[B62] Defazio G. Berardelli A. Fabbrini G. Martino D. Fincati E. Fiaschi A. . (2008). Pain as a nonmotor symptom of Parkinson disease: evidence from a case-control study. Arch. Neurol. 65, 1191–1194. doi: 10.1001/archneurol.2008.2 18779422

[B63] Dellarole I. L. Vacchi E. Ruiz-Barrio I. Pinton S. Raimondi A. Rossi S. . (2024). Tau seeding activity in skin biopsy differentiates tauopathies from synucleinopathies. NPJ Parkinsons Dis. 10:116. doi: 10.1038/s41531-024-00728-9 38879633 PMC11180195

[B64] Devanand D. P. Michaels-Marston K. S. Liu X. Pelton G. H. Padilla M. Marder K. . (2000). Olfactory deficits in patients with mild cognitive impairment predict Alzheimer's disease at follow-up. Am. J. Psychiatry 157, 1399–1405. doi: 10.1176/appi.ajp.157.9.1399 10964854

[B65] Devenney E. Vucic S. Hodges J. R. Kiernan M. C. (2015). Motor neuron disease-frontotemporal dementia: a clinical continuum. Expert Rev. Neurother. 15, 509–522. doi: 10.1586/14737175.2015.1034108 25865485

[B66] Devi Y. M. Kumar M. Tiwari A. Dhar M. Kumari S. Kumar N. (2025). Comparison of clinical and electrophysiologic characteristics of peripheral neuropathy in progressive supranuclear palsy and parkinson's disease: an observational study. Ann. Indian Acad. Neurol. 28, 72–78. doi: 10.4103/aian.aian_512_24 39639527 PMC11892956

[B67] Devigili G. Cazzato D. Lauria G. (2020). Clinical diagnosis and management of small fiber neuropathy: an update on best practice. Expert Rev. Neurother. 20, 967–980. doi: 10.1080/14737175.2020.1794825 32654574

[B68] Djaldetti R. Shifrin A. Rogowski Z. Sprecher E. Melamed E. Yarnitsky D. (2004). Quantitative measurement of pain sensation in patients with Parkinson disease. Neurology 62, 2171–2175. doi: 10.1212/01.WNL.0000130455.38550.9D 15210877

[B69] Donadio V. Incensi A. Leta V. Giannoccaro M. P. Scaglione C. Martinelli P. . (2014). Skin nerve α-synuclein deposits: a biomarker for idiopathic Parkinson disease. Neurology 82, 1362–1369. doi: 10.1212/WNL.0000000000000316 24634456

[B70] Donadio V. Incensi A. Rizzo G. De Micco R. Tessitore A. Devigili G. . (2020). Skin biopsy may help to distinguish multiple system atrophy-Parkinsonism from Parkinson's disease with orthostatic hypotension. Mov. Disord. 35, 1649–1657. doi: 10.1002/mds.28126 32557839

[B71] Donadio V. Incensi A. Rizzo G. Westermark G. T. Devigili G. De Micco R. . (2023). Phosphorylated α-synuclein in skin Schwann cells: a new biomarker for multiple system atrophy. Brain 146, 1065–1074. doi: 10.1093/brain/awac124 35552610

[B72] Doppler K. Ebert S. Uçeyler N. Trenkwalder C. Ebentheuer J. Volkmann J. . (2014). Cutaneous neuropathy in Parkinson's disease: a window into brain pathology. Acta Neuropathol. 128, 99–109. doi: 10.1007/s00401-014-1284-0 24788821 PMC4059960

[B73] Dubbioso R. Provitera V. Vitale F. Stancanelli A. Borreca I. Caporaso G. . (2021). Cutaneous sensory and autonomic denervation in progressive supranuclear palsy. Neuropathol. Appl. Neurobiol. 47, 653–663. doi: 10.1111/nan.12692 33421177

[B74] Dugger B. N. Harvey D. Beach T. G. Adler C. H. (2022). Peripheral tau as a biomarker for neurodegenerative diseases: is life on Earth, life on Mars? Brain 145, 2629–2631. doi: 10.1093/brain/awac281 35947169 PMC9420015

[B75] Dugger B. N. Hoffman B. R. Scroggins A. Serrano G. E. Adler C. H. Shill H. A. . (2019). Tau immunoreactivity in peripheral tissues of human aging and select tauopathies. Neurosci. Lett. 696, 132–139. doi: 10.1016/j.neulet.2018.12.031 30579993 PMC7357994

[B76] Dyck P. J. Kratz K. M. Karnes J. L. Litchy W. J. Klein R. Pach J. M. . (1993). The prevalence by staged severity of various types of diabetic neuropathy, retinopathy, and nephropathy in a population-based cohort: the Rochester Diabetic Neuropathy Study. Neurology 43, 817–824. doi: 10.1212/WNL.43.4.817 8469345

[B77] Egenolf N. Zu Altenschildesche C. M. Kreß L. Eggermann K. Namer B. Gross F. . (2021). Diagnosing small fiber neuropathy in clinical practice: a deep phenotyping study. Ther. Adv. Neurol. Disord. 14:17562864211004318. doi: 10.1177/17562864211004318 34335876 PMC8283814

[B78] England J. D. Gronseth G. S. Franklin G. Carter G. T. Kinsella L. J. Cohen J. A. . (2009). Practice parameter: evaluation of distal symmetric polyneuropathy: role of laboratory and genetic testing (an evidence-based review). Report of the American Academy of Neurology, American Association of Neuromuscular and Electrodiagnostic Medicine, and American Academy of Physical Medicine and Rehabilitation. Neurology 72, 185–192. doi: 10.1212/01.wnl.0000336370.51010.a1 19056666

[B79] Erro M. E. Arrondo P. Gastón I. Clavero P. de Gordoa J. S. R. Martí Andrés G. . (2024). Epidemiologic and clinical features of multisystem atrophy: a population-based study in Navarre, Spain. J. Neurol. 271, 6647–6654. doi: 10.1007/s00415-024-12561-4 39136751 PMC11446993

[B80] Espay A. J. Herrup K. Kepp K. P. Daly T. (2023). The proteinopenia hypothesis: Loss of Aβ and the onset of Alzheimer's Disease. Ageing Res. Rev. 92:102112. doi: 10.1016/j.arr.2023.102112 38270185

[B604] Fang Y. Jin C. Y. Zheng R. Wu J. M. Zhang B. R. Pu J. L. (2021). Asymmetric, multifocal musculoskeletal pain preceding the onset of progressive supranuclear palsy: a case report. CNS Neurosci. Ther. 27, 256–258. doi: 10.1111/cns.13527 PMC7816200 33274849

[B81] Farhad K. Traub R. Ruzhansky K. M. Brannagan T. H. (2016). 3rd. Causes of neuropathy in patients referred as “idiopathic neuropathy”. Muscle Nerve. 53, 856–861. doi: 10.1002/mus.24969 26561790

[B82] Feldman E. L. Callaghan B. C. Pop-Busui R. Zochodne D. W. Wright D. E. Bennett D. L. . (2019). Diabetic neuropathy. Nat. Rev. Dis. Primers 5:42. doi: 10.1038/s41572-019-0092-1 31197183 PMC7096070

[B83] Feldman E. L. Goutman S. A. Petri S. Mazzini L. Savelieff M. G. Shaw P. J. . (2022). Amyotrophic lateral sclerosis. Lancet 400, 1363–1380. doi: 10.1016/S0140-6736(22)01272-7 36116464 PMC10089700

[B84] Fletcher P. D. Downey L. E. Golden H. L. Clark C. N. Slattery C. F. Paterson R. W. (2015) Pain temperature processing in dementia: a clinical neuroanatomical analysis. Brain 138(Pt 11), 3360–3372. doi: 10.1093/brain/awv276. 26463677 PMC4620514

[B85] Foesleitner O. Knop K. C. Lindenau M. Preisner F. Bäumer P. Heiland S. . (2023). Quantitative MR neurography in multifocal motor neuropathy and amyotrophic lateral sclerosis. Diagnostics 13:1237. doi: 10.3390/diagnostics13071237 37046455 PMC10093201

[B86] Foiani M. S. Woollacott I. O. Heller C. Bocchetta M. Heslegrave A. Dick K. M. . (2018). Plasma tau is increased in frontotemporal dementia. J. Neurol. Neurosurg. Psychiatry 89, 804–807. doi: 10.1136/jnnp-2017-317260 29440230 PMC6204947

[B87] Frank S. (2014). Treatment of Huntington's disease. Neurotherapeutics 11, 153–160. doi: 10.1007/s13311-013-0244-z 24366610 PMC3899480

[B88] Freeman R. Gewandter J. S. Faber C. G. Gibbons C. Haroutounian S. Lauria G. . (2020). Idiopathic distal sensory polyneuropathy: ACTTION diagnostic criteria. Neurology 95, 1005–1014. doi: 10.1212/WNL.0000000000010988 33055271 PMC7734920

[B89] Fu J. He J. Zhang Y. Liu Z. Wang H. Li J. . (2022). Small fiber neuropathy for assessment of disease severity in amyotrophic lateral sclerosis: corneal confocal microscopy findings. Orphanet J. Rare Dis. 17:7. doi: 10.1186/s13023-021-02157-w 34991673 PMC8734326

[B90] Gad H. Kalra S. Pinzon R. Garcia R. N. Yotsombut K. Coetzee A. . (2024). Earlier diagnosis of peripheral neuropathy in primary care: a call to action. J. Peripher. Nerv. Syst. 29, 28–37. doi: 10.1111/jns.12613 38268316

[B91] Galassi G. Nemni R. Baraldi A. Gibertoni M. Colombo A. (1982). Peripheral neuropathy in multiple system atrophy with autonomic failure. Neurology 32, 1116–1121. doi: 10.1212/WNL.32.10.1116 6289187

[B92] Geber C. Breimhorst M. Burbach B. Egenolf C. Baier B. Fechir M. . (2013). Pain in chemotherapy-induced neuropathy–more than neuropathic? Pain 154, 2877–2887. doi: 10.1016/j.pain.2013.08.028 23999056

[B93] Gemignani F. Bellanova M. F. Saccani E. Pavesi G. (2022). Non-length-dependent small fiber neuropathy: not a matter of stockings and gloves. Muscle Nerve 65, 10–28. doi: 10.1002/mus.27379 34374103

[B94] Gentile F. Scarlino S. Falzone Y. M. Lunetta C. Tremolizzo L. Quattrini A. . (2019). The peripheral nervous system in amyotrophic lateral sclerosis: opportunities for translational research. Front. Neurosci. 13:601. doi: 10.3389/fnins.2019.00601 31293369 PMC6603245

[B95] Ghosh S. Lippa C. F. (2015). Clinical subtypes of frontotemporal dementia. Am. J. Alzheimers Dis. Other Demen. 30, 653–661. doi: 10.1177/1533317513494442 23813692 PMC10852906

[B96] Gibbons C. Wang N. Rajan S. Kern D. Palma J. A. Kaufmann H. . (2023). Cutaneous α-synuclein signatures in patients with multiple system atrophy and parkinson disease. Neurology 100, e1529–e39. doi: 10.1212/WNL.0000000000206772 36657992 PMC10103107

[B97] Ginsberg L. (2020). Acute and chronic neuropathies. Medicine 48, 612–618. doi: 10.1016/j.mpmed.2020.06.009 32837227 PMC7382348

[B98] Gloor Y. Matthey A. Sobo K. Mouterde M. Kosek E. Pickering G. . (2022). Uncovering a genetic polymorphism located in huntingtin associated protein 1 in modulation of central pain sensitization signaling pathways. Front. Neurosci. 16:807773. doi: 10.3389/fnins.2022.807773 35837121 PMC9274135

[B99] Goh Y. Y. Saunders E. Pavey S. Rushton E. Quinn N. Houlden H. . (2023). Multiple system atrophy. Pract Neurol. 23, 208–21. doi: 10.1136/pn-2020-002797 36927875 PMC10314081

[B100] Gomatos E. L. Dulebohn S. C. Rehman A. (2025). Sensory Neuropathy. StatPearls. Treasure Island, FL: StatPearls Publishing. 32644446

[B101] Gonzalez-Ortiz F. Karikari T. K. Bentivenga G. M. Baiardi S. Mammana A. Turton M. . (2024). Levels of plasma brain-derived tau and p-tau181 in Alzheimer's disease and rapidly progressive dementias. Alzheimers Dement. 20, 745–751. doi: 10.1002/alz.13516 37858957 PMC10841678

[B102] Grijalvo-Perez A. M. Litvan I. (2014). Corticobasal degeneration. Semin Neurol. 34, 160–173. doi: 10.1055/s-0034-1381734 24963675

[B103] Guo L. Duggan J. Cordeiro M. F. (2010). Alzheimer's disease and retinal neurodegeneration. Curr. Alzheimer Res. 7, 3–14. doi: 10.2174/156720510790274491 20205667

[B104] Gwathmey K. G. (2016). Sensory neuronopathies. Muscle Nerve 53, 8–19. doi: 10.1002/mus.24943 26467754

[B105] Hameed S. Cascella M. (2025). Multifocal Motor Neuropathy. StatPearls. Treasure Island, FL: StatPearls Publishing. 32119411

[B106] Hammad M. Silva A. Glass J. Sladky J. T. Benatar M. (2007). Clinical, electrophysiologic, and pathologic evidence for sensory abnormalities in ALS. Neurology 69, 2236–2242. doi: 10.1212/01.wnl.0000286948.99150.16 18071143

[B107] Hammi C. Yeung B. (2025). Neuropathy. StatPearls. Treasure Island, FL: StatPearls Publishing.

[B108] Handy C. R. Krudy C. Boulis N. Federici T. (2011). Pain in amyotrophic lateral sclerosis: a neglected aspect of disease. Neurol. Res. Int. 2011:403808. doi: 10.1155/2011/403808 21766021 PMC3135011

[B109] Hanewinckel R. Drenthen J. van Oijen M. Hofman A. van Doorn P. A. Ikram M. A. (2016a). Prevalence of polyneuropathy in the general middle-aged and elderly population. Neurology 87, 1892–1898. doi: 10.1212/WNL.0000000000003293 27683845

[B110] Hanewinckel R. Ikram M. A. Van Doorn P. A. (2016b). Peripheral neuropathies. Handb. Clin. Neurol. 138, 263–282. doi: 10.1016/B978-0-12-802973-2.00015-X 27637963

[B111] Hayward L. Zubrick S. R. Hall W. (1985). Early sensory-perceptual changes in Huntington's disease. Aust. N. Z. J. Psychiatry 19, 384–389. doi: 10.1080/00048678509158846 2938570

[B112] Hicks C. W. Wang D. Windham B. G. Matsushita K. Selvin E. (2021). Prevalence of peripheral neuropathy defined by monofilament insensitivity in middle-aged and older adults in two US cohorts. Sci. Rep. 11:19159. doi: 10.1038/s41598-021-98565-w 34580377 PMC8476511

[B113] Höglinger G. U. Respondek G. Stamelou M. Kurz C. Josephs K. A. Lang A. E. . (2017). Clinical diagnosis of progressive supranuclear palsy: the movement disorder society criteria. Mov Disord. 32, 853–864. doi: 10.1002/mds.26987 28467028 PMC5516529

[B114] Iglesias C. Sangari S. El Mendili M. M. Benali H. Marchand-Pauvert V. Pradat P. F. (2015). Electrophysiological and spinal imaging evidences for sensory dysfunction in amyotrophic lateral sclerosis. BMJ Open 5:e007659. doi: 10.1136/bmjopen-2015-007659 25712823 PMC4342589

[B115] Ilieva H. Vullaganti M. Kwan J. (2023). Advances in molecular pathology, diagnosis, and treatment of amyotrophic lateral sclerosis. BMJ 383:e075037. doi: 10.1136/bmj-2023-075037 37890889 PMC10603569

[B116] Isaacs J. D. Dean A. F. Shaw C. E. Al-Chalabi A. Mills K. R. Leigh P. N. (2007). Amyotrophic lateral sclerosis with sensory neuropathy: part of a multisystem disorder? J. Neurol. Neurosurg. Psychiatry 78, 750–753. doi: 10.1136/jnnp.2006.098798 17575021 PMC2117693

[B117] Isak B. Pugdahl K. Karlsson P. Tankisi H. Finnerup N. B. Furtula J. . (2017). Quantitative sensory testing and structural assessment of sensory nerve fibres in amyotrophic lateral sclerosis. J. Neurol. Sci. 373, 329–334. doi: 10.1016/j.jns.2017.01.005 28131214

[B118] Islam M. N. Maeda N. Miyasato E. Jahan M. R. Tarif A. M. M. Ishino T. . (2020). Expression of huntingtin-associated protein 1 in adult mouse dorsal root ganglia and its neurochemical characterization in reference to sensory neuron subpopulations. IBRO Rep. 9, 258–269. doi: 10.1016/j.ibror.2020.10.001 33089002 PMC7560692

[B119] Jankovic J. Tan E. K. (2020). Parkinson's disease: etiopathogenesis and treatment. J. Neurol. Neurosurg. Psychiatr. 91, 795–808. doi: 10.1136/jnnp-2019-322338 32576618

[B120] Javed S. Petropoulos I. N. Alam U. Malik R. A. (2015). Treatment of painful diabetic neuropathy. Ther. Adv. Chronic Dis. 6, 15–28. doi: 10.1177/2040622314552071 25553239 PMC4269610

[B509] Jensen-Dahm C. Werner M. U. Dahl J. B. Jensen T. S. Ballegaard M. Hejl A. M. . (2014). Quantitative sensory testing and pain tolerance in patients with mild to moderate Alzheimer disease compared to healthy control subjects. Pain 155, 1439–1445. doi: 10.1016/j.pain.2013.12.031 24412285

[B121] Jeziorska M. Atkinson A. Kass-Iliyya L. Javed S. Kobylecki C. Gosal D. . (2019a). Increased intraepidermal nerve fiber degeneration and impaired regeneration relate to symptoms and deficits in Parkinson's disease. Front. Neurol. 10:111. doi: 10.3389/fneur.2019.00111 30837937 PMC6383044

[B122] Jeziorska M. Atkinson A. Kass-Iliyya L. Kobylecki C. Gosal D. Marshall A. . (2019b). Small fibre neuropathy in Parkinson's disease: comparison of skin biopsies from the more affected and less affected sides. J. Parkinsons. Dis. 9, 761–765. doi: 10.3233/JPD-191697 31381529 PMC6839493

[B123] Jin H. Y. Lee K. A. Kim Y. J. Gwak I. S. Park T. S. Yeom S. W. . (2023). Bidirectional association between diabetic peripheral neuropathy and vitamin B12 deficiency: two longitudinal 9-year follow-up studies using a national sample cohort. Prim. Care Diabetes. 17, 436–443. doi: 10.1016/j.pcd.2023.06.006 37344286

[B124] Jin Y. Wang W. Chen W. Guo S. Li C. Zhu D. . (2021). Corneal confocal microscopy: a useful tool for diagnosis of small fiber neuropathy in type 2 diabetes. J. Diabetes Investig. 12, 2183–2189. doi: 10.1111/jdi.13616 34134175 PMC8668068

[B125] Jo M. Lee S. Jeon Y. M. Kim S. Kwon Y. Kim H. J. (2020). The role of TDP-43 propagation in neurodegenerative diseases: integrating insights from clinical and experimental studies. Exp. Mol. Med. 52, 1652–1662. doi: 10.1038/s12276-020-00513-7 33051572 PMC8080625

[B126] Karve S. J. Ringman J. M. Lee A. S. Juarez K. O. Mendez M. F. (2012). Comparison of clinical characteristics between familial and non-familial early onset Alzheimer's disease. J. Neurol. 259, 2182–2188. doi: 10.1007/s00415-012-6481-y 22460587 PMC3442121

[B127] Kass-Iliyya L. Javed S. Gosal D. Kobylecki C. Marshall A. Petropoulos I. N. . (2015a). Small fiber neuropathy in Parkinson's disease: a clinical, pathological and corneal confocal microscopy study. Parkinsonism Relat .Disord. 21, 1454–1460. doi: 10.1016/j.parkreldis.2015.10.019 26578039 PMC4671992

[B128] Kass-Iliyya L. Kobylecki C. McDonald K. R. Gerhard A. Silverdale M. A. (2015b). Pain in multiple system atrophy and progressive supranuclear palsy compared to Parkinson's disease. Brain Behav. 5:e00320. doi: 10.1002/brb3.320 25874161 PMC4389053

[B129] Kauppila L. A. Ten Holter S. E. M. van de Warrenburg B. Bloem B. R. (2022). A guide for the differential diagnosis of multiple system atrophy in clinical practice. J Parkinsons Dis. 12, 2015–2027. doi: 10.3233/JPD-223392 36057832 PMC9661336

[B130] Kazamel M. Stino A. M. Smith A. G. (2021). Metabolic syndrome and peripheral neuropathy. Muscle Nerve 63, 285–293. doi: 10.1002/mus.27086 33098165

[B131] Kirthi V. Perumbalath A. Brown E. Nevitt S. Petropoulos I. N. Burgess J. . (2021). Prevalence of peripheral neuropathy in pre-diabetes: a systematic review. BMJ Open Diabetes Res. Care 9:e002040. doi: 10.1136/bmjdrc-2020-002040 34006607 PMC8137250

[B132] Kleinerova J. Chipika R. H. Tan E. L. Yunusova Y. Marchand-Pauvert V. Kassubek J. . (2025). Sensory dysfunction in ALS and other motor neuron diseases: clinical relevance histopathology, neurophysiology and insights from neuroimaging. Biomedicines 13:559. doi: 10.3390/biomedicines13030559 40149536 PMC11940395

[B133] Koga S. Aoki N. Uitti R. J. van Gerpen J. A. Cheshire W. P. Josephs K. A. . (2015). When DLB, PD, and PSP masquerade as MSA: an autopsy study of 134 patients. Neurology 85, 404–412. doi: 10.1212/WNL.0000000000001807 26138942 PMC4534078

[B508] Koga S. Josephs K. A. Aiba I. Yoshida M. Dickson D. W. (2022). Neuropathology and emerging biomarkers in corticobasal syndrome. J. Neurol. Neurosurg. Psychiatry 93, 919–929. doi: 10.1136/jnnp-2021-328586 PMC9380481 35697501

[B134] Koga S. Kouri N. Walton R. L. Ebbert M. T. W. Josephs K. A. Litvan I. . (2018). Corticobasal degeneration with TDP-43 pathology presenting with progressive supranuclear palsy syndrome: a distinct clinicopathologic subtype. Acta Neuropathol. 136, 389–404. doi: 10.1007/s00401-018-1878-z 29926172 PMC6309287

[B135] Koller W. C. (1984). Sensory symptoms in Parkinson's disease. Neurology 34, 957–959. doi: 10.1212/WNL.34.7.957 6539873

[B136] Kompoliti K. Goetz C. G. Boeve B. F. Maraganore D. M. Ahlskog J. E. Marsden C. D. . (1998). Clinical presentation and pharmacological therapy in corticobasal degeneration. Arch. Neurol. 55, 957–961. doi: 10.1001/archneur.55.7.957 9678313

[B137] Koppisetti R. K. Barthélemy N. R. Horie K. Ly C. V. Roberts K. F. Koutarapu S. . (2025). Distribution of big tau isoforms in the human central and peripheral nervous system. bioRxiv. [preprint]. doi: 10.1101/2025.09.15.676127 42473039 PMC13587115

[B138] Kouli A. Torsney K. M. Kuan W. L. (2018). Parkinson's disease: etiology, neuropathology, and pathogenesis. In: Stoker TB, Greenland JC, editors. Parkinson's Disease: Pathogenesis and Clinical Aspects. Brisbane, AU: Codon Publications. 30702842

[B139] Kouri N. Whitwell J. L. Josephs K. A. Rademakers R. Dickson D. W. (2011). Corticobasal degeneration: a pathologically distinct 4R tauopathy. Nat. Rev. Neurol. 7, 263–272. doi: 10.1038/nrneurol.2011.43 21487420 PMC10006729

[B140] Kovacs G. G. Lukic M. J. Irwin D. J. Arzberger T. Respondek G. Lee E. B. . (2020). Distribution patterns of tau pathology in progressive supranuclear palsy. Acta Neuropathol. 140, 99–119. doi: 10.1007/s00401-020-02158-2 32383020 PMC7360645

[B141] Krämer H. H. Rebhorn C. Geber C. Birklein F. (2021). Sympathetic and sensory nerve fiber function in multiple system atrophy and idiopathic Parkinson's disease. J. Neurol. 268, 3435–3443. doi: 10.1007/s00415-021-10514-9 33715046 PMC8357748

[B142] Krismer F. Fanciulli A. Meissner W. G. Coon E. A. Wenning G. K. (2024). Multiple system atrophy: advances in pathophysiology, diagnosis, and treatment. Lancet Neurol. 23, 1252–1266. doi: 10.1016/S1474-4422(24)00396-X 39577925

[B143] Kumaradev S. Fayosse A. Dugravot A. Dumurgier J. Roux C. Kivimäki M. . (2021). Timeline of pain before dementia diagnosis: a 27-year follow-up study. Pain 162, 1578–1585. doi: 10.1097/j.pain.0000000000002080 33003109 PMC7985036

[B144] Kuzdas-Wood D. Irschick R. Theurl M. Malsch P. Mair N. Mantinger C. . (2015). Involvement of peripheral nerves in the transgenic PLP-α-syn model of multiple system atrophy: extending the phenotype. PLoS ONE 10:e0136575. doi: 10.1371/journal.pone.0136575 26496712 PMC4619736

[B145] Kwak S. (2022). Pain in amyotrophic lateral sclerosis: a narrative review. J. Yeungnam Med. Sci. 39, 181–189. doi: 10.12701/jyms.2022.00332 35673830 PMC9273136

[B146] Lacomis D. (2002). Small-fiber neuropathy. Muscle Nerve 26, 173–188. doi: 10.1002/mus.10181 12210380

[B147] Lama J. Salabasidou E. Volkmann J. Kuzkina A. Duty S. Üçeyler N. (2022). Proinflammatory profile in the skin of Parkinson's disease patients with and without pain. PLoS ONE 17:e0276564. doi: 10.1371/journal.pone.0276564 36301901 PMC9612575

[B148] Lamb R. Rohrer J. D. Lees A. J. Morris H. R. (2016). Progressive supranuclear palsy and corticobasal degeneration: pathophysiology and treatment options. Curr. Treat Options Neurol. 18:42. doi: 10.1007/s11940-016-0422-5 27526039 PMC4985534

[B149] Landqvist Waldö M. Santillo A. F. Gustafson L. Englund E. Passant U. (2014). Somatic complaints in frontotemporal dementia. Am. J. Neurodegener. Dis. 3, 84–92. 25232513 PMC4162589

[B150] Leblhuber F. Schroecksnadel K. Beran-Praher M. Haller H. Steiner K. Fuchs D. (2011). Polyneuropathy and dementia in old age: common inflammatory and vascular parameters. J. Neural Transm. 118, 721–725. doi: 10.1007/s00702-011-0579-8 21286925

[B151] Lehmann H. C. Wunderlich G. Fink G. R. Sommer C. (2020). Diagnosis of peripheral neuropathy. Neurol Res Pract. 2:20. doi: 10.1186/s42466-020-00064-2 33324924 PMC7650053

[B152] Lenka A. Jankovic J. (2025). Corticobasal syndrome: are there central or peripheral triggers? Neurol. Clin. Pract. 15:e200365. doi: 10.1212/CPJ.0000000000200365 39399563 PMC11464233

[B153] Li J. Wang Y. Yang R. Ma W. Yan J. Li Y. . (2023). Pain in Huntington's disease and its potential mechanisms. Front. Aging Neurosci. 15:1190563. doi: 10.3389/fnagi.2023.1190563 37484692 PMC10357841

[B154] Lim S. H. Ferdousi M. Bhattacharjee S. Kalteniece A. Mahfoud Z. R. Petropoulos I. N. . (2023). Corneal confocal microscopy demonstrates varying degrees of neurodegeneration in atypical parkinsonian disorders. Parkinsonism Relat. Disord. 117:105899. doi: 10.1016/j.parkreldis.2023.105899 37948830

[B155] Lin Y. J. Kao T. W. Chen W. L. (2021). Relationship between peripheral neuropathy and cognitive performance in the elderly population. Medicine 100:e26071. doi: 10.1097/MD.0000000000026071 34011128 PMC8137106

[B156] Liu J. Zhang X. Ding X. Song M. Sui K. (2019). Analysis of clinical and electrophysiological characteristics of 150 patients with amyotrophic lateral sclerosis in China. Neurol. Sci. 40, 363–369. doi: 10.1007/s10072-018-3633-6 30467636

[B157] Liu M. Liu R. Yuan Y. Liu X. Li L. Wang Y. . (2025). Identification of small fiber neuropathy in neuronal intranuclear inclusion disease: a clinicopathological study. Alzheimer Dement. 21:e14596. doi: 10.1002/alz.14596 39988644 PMC11847649

[B158] Liu R. Durbin-Johnson B. Paciotti B. Liu A. T. Weakley A. Liu X. . (2024). Metabolic dysfunctions predict the development of Alzheimer's disease: statistical and machine learning analysis of EMR data. Alzheimers Dement. 20, 6765–6775. doi: 10.1002/alz.14101 39140368 PMC11485292

[B159] Lopes B. Sousa P. Alvites R. Branquinho M. Sousa A. C. Mendonça C. . (2022). Peripheral Nerve injury treatments and advances: one health perspective. Int. J. Mol. Sci. 23:918. doi: 10.3390/ijms23020918 35055104 PMC8779751

[B160] Lowy D. B. Makker P. G. S. Moalem-Taylor G. (2021). Cutaneous neuroimmune interactions in peripheral neuropathic pain states. Front. Immunol. 12:660203. doi: 10.3389/fimmu.2021.660203 33912189 PMC8071857

[B161] Lyons S. Trépel D. Lynch T. Walsh R. O'Dowd S. (2023). The prevalence and incidence of progressive supranuclear palsy and corticobasal syndrome: a systematic review and meta-analysis. J. Neurol. 270, 4451–4465. doi: 10.1007/s00415-023-11791-2 37289323 PMC10421779

[B162] Maccioni R. B. Farías G. Morales I. Navarrete L. (2010). The revitalized tau hypothesis on Alzheimer's disease. Arch. Med. Res. 41, 226–231. doi: 10.1016/j.arcmed.2010.03.007 20682182

[B163] Maccioni R. B. Rojo L. E. Fernández J. A. Kuljis R. O. (2009). The role of neuroimmunomodulation in Alzheimer's disease. Ann. N. Y. Acad. Sci. 1153, 240–246. doi: 10.1111/j.1749-6632.2008.03972.x 19236346

[B164] Machado-Costa M. C. Mitne-Neto M. Costa L. H. D. Alves L. M. Oliveira A. S. B. Zatz M. . (2023). Small-fibre neuropathy in patients with familial amyotrophic lateral sclerosis type 8. Can. J. Neurol. Sci. 50, 885–890. doi: 10.1017/cjn.2022.331 36457144

[B165] Mahale R. R. Krishnan S. Divya K. P. Jisha V. T. Kishore A. (2021). Subtypes of PSP and prognosis: a retrospective analysis. Ann. Indian Acad. Neurol. 24, 56–62. doi: 10.4103/aian.AIAN_611_20 33911380 PMC8061531

[B166] Mancini F. Comi C. Oggioni G. D. Pacchetti C. Calandrella D. Coletti Moja M. . (2014). Prevalence and features of peripheral neuropathy in Parkinson's disease patients under different therapeutic regimens. Parkinsonism Relat. Disord. 20, 27–31. doi: 10.1016/j.parkreldis.2013.09.007 24099722

[B167] Marchettini P. Lacerenza M. Mauri E. Marangoni C. (2006). Painful peripheral neuropathies. Curr. Neuropharmacol. 4, 175–181. doi: 10.2174/157015906778019536 18615140 PMC2430688

[B168] Marques Sousa C. Humbert S. (2013). Huntingtin: here, there, everywhere! *J Huntingtons Dis*. 2, 395–403. doi: 10.3233/JHD-130082 25062728

[B169] Marques N. Azevedo O. Almeida A. R. Bento D. Cruz I. Correia E. . (2020). Specific therapy for transthyretin cardiac amyloidosis: a systematic literature review and evidence-based recommendations. J. Am. Heart Assoc. 9:e016614. doi: 10.1161/JAHA.120.016614 32969287 PMC7792401

[B170] Marquez A. Guernsey L. S. Frizzi K. E. Cundiff M. Constantino I. Muttalib N. . (2021). Tau associated peripheral and central neurodegeneration: identification of an early imaging marker for tauopathy. Neurobiol. Dis. 151:105273. doi: 10.1016/j.nbd.2021.105273 33482356 PMC7925437

[B171] Marshall C. R. Hardy C. J. D. Russell L. L. Bond R. L. Sivasathiaseelan H. Greaves C. . (2019). The functional neuroanatomy of emotion processing in frontotemporal dementias. Brain 142, 2873–2887. doi: 10.1093/brain/awz204 31321407 PMC7959336

[B172] Martin C. L. Albers J. W. Pop-Busui R. (2014). Neuropathy and related findings in the diabetes control and complications trial/epidemiology of diabetes interventions and complications study. Diabetes Care 37, 31–38. doi: 10.2337/dc13-2114 24356595 PMC3868000

[B173] Masrori P. Van Damme P. (2020). Amyotrophic lateral sclerosis: a clinical review. Eur. J. Neurol. 27, 1918–1929. doi: 10.1111/ene.14393 32526057 PMC7540334

[B174] McKay J. H. Cheshire W. P. (2018). First symptoms in multiple system atrophy. Clin. Auton. Res. 28, 215–221. doi: 10.1007/s10286-017-0500-0 29313153 PMC5859695

[B175] Meade R. M. Fairlie D. P. Mason J. M. (2019). Alpha-synuclein structure and Parkinson's disease - lessons and emerging principles. Mol. Neurodegener. 14:29. doi: 10.1186/s13024-019-0329-1 31331359 PMC6647174

[B176] Medina A. Mahjoub Y. Shaver L. Pringsheim T. (2022). Prevalence and incidence of Huntington's disease: an updated systematic review and meta-analysis. Mov. Disord. 37, 2327–2335. doi: 10.1002/mds.29228 36161673 PMC10086981

[B177] Mehta P. Raymond J. Zhang Y. Punjani R. Han M. Larson T. . (2023). Prevalence of amyotrophic lateral sclerosis in the United States, 2018. *Amyotroph. Lateral Scler. Frontotemporal*. Degener. 24, 702–708. doi: 10.1080/21678421.2023.2245858 37602649

[B178] Melli G. Vacchi E. Biemmi V. Galati S. Staedler C. Ambrosini R. . (2018). Cervical skin denervation associates with alpha-synuclein aggregates in Parkinson disease. Ann. Clin. Transl. Neurol. 5, 1394–1407. doi: 10.1002/acn3.669 30480033 PMC6243385

[B179] Mellone M. Kestoras D. Andrews M. R. Dassie E. Crowther R. A. Stokin G. B. . (2013). Tau pathology is present *in vivo* and develops *in vitro* in sensory neurons from human P301S tau transgenic mice: a system for screening drugs against tauopathies. J. Neurosci. 33, 18175–18189. doi: 10.1523/JNEUROSCI.4933-12.2013 24227726 PMC3828468

[B601] Merlo S. Costa L. Chiechio S. Busceti C. L. Ciranna L. Santangelo R. . (2023). Increased heat pain tolerance but hyperalgesia to tonic inflammatory pain in the CRND8 mouse model of Alzheimer's disease. J. Alzheimers Dis. 96, 77–91. doi: 10.3233/JAD-230148 PMC10657672 37742639

[B180] Mian M. Salehi F. Patel R. Tahiri J. Bel-Hadj-Kacem A. Alhaque A. . (2025). Exploring the cognitive impacts of diabetic neuropathy: a comprehensive review. Biochim. Biophys. Acta Mol. Basis Dis. 1871:167892. doi: 10.1016/j.bbadis.2025.167892 40324733

[B181] Miklossy J. Qing H. Radenovic A. Kis A. Vileno B. Làszló F. . (2010). Beta amyloid and hyperphosphorylated tau deposits in the pancreas in type 2 diabetes. Neurobiol. Aging 31, 1503–1515. doi: 10.1016/j.neurobiolaging.2008.08.019 18950899 PMC4140193

[B182] Mirian A. Aljohani Z. Grushka D. Florendo-Cumbermack A. (2023). Diagnosis and management of patients with polyneuropathy. Cmaj 195, E227–e33. doi: 10.1503/cmaj.220936 36781195 PMC9928442

[B183] Misra U. K. Kalita J. Nair P. P. (2008). Diagnostic approach to peripheral neuropathy. Ann. Indian Acad. Neurol. 11, 89–97. doi: 10.4103/0972-2327.41875 19893645 PMC2771953

[B184] Moberg P. J. Pearlson G. D. Speedie L. J. Lipsey J. R. Strauss M. E. Folstein S. E. (1987). Olfactory recognition: differential impairments in early and late Huntington's and Alzheimer's diseases. J. Clin. Exp. Neuropsychol. 9, 650–664. doi: 10.1080/01688638708405208 2961789

[B185] Moser A. D. Epping E. Espe-Pfeifer P. Martin E. Zhorne L. Mathews K. . (2017). A survey-based study identifies common but unrecognized symptoms in a large series of juvenile Huntington's disease. Neurodegener. Dis. Manag. 7, 307–315. doi: 10.2217/nmt-2017-0019 29043929

[B186] Mostofi A. Morgante F. Edwards M. J. Brown P. Pereira E. A. C. (2021). Pain in Parkinson's disease and the role of the subthalamic nucleus. Brain 144, 1342–1350. doi: 10.1093/brain/awab001 34037696 PMC7612468

[B187] Moustafa A. A. Chakravarthy S. Phillips J. R. Gupta A. Keri S. Polner B. . (2016). Motor symptoms in Parkinson's disease: a unified framework. Neurosci. Biobehav. Rev. 68, 727–740. doi: 10.1016/j.neubiorev.2016.07.010 27422450

[B188] Nègre-Pagès L. Regragui W. Bouhassira D. Grandjean H. Rascol O. (2008). Chronic pain in Parkinson's disease: the cross-sectional French DoPaMiP survey. Mov. Disord. 23, 1361–1369. doi: 10.1002/mds.22142 18546344

[B189] Nijs M. Van Damme P. (2024). The genetics of amyotrophic lateral sclerosis. Curr. Opin. Neurol. 37, 560–569. doi: 10.1097/WCO.0000000000001294 38967083 PMC11377058

[B190] Niu X. Yin P. Guan C. Shao Q. Cui G. Zan K. . (2024). Corneal confocal microscopy may help to distinguish Multiple System Atrophy from Parkinson's disease. NPJ Parkinsons Dis. 10:63. doi: 10.1038/s41531-024-00680-8 38493181 PMC10944503

[B191] Nolano M. Caporaso G. Manganelli F. Stancanelli A. Borreca I. Mozzillo S. . (2022). Phosphorylated α-synuclein deposits in cutaneous nerves of early Parkinsonism. J. Parkinsons Dis. 12, 2453–2468. doi: 10.3233/JPD-223421 36373295

[B192] Nolano M. Provitera V. Caporaso G. Fasolino I. Borreca I. Stancanelli A. . (2024). Skin innervation across amyotrophic lateral sclerosis clinical stages: new prognostic biomarkers. Brain 147, 1740–1750. doi: 10.1093/brain/awad426 38123494

[B193] Nolano M. Provitera V. Stancanelli A. Saltalamacchia A. M. Caporaso G. Lullo F. . (2018). Small fiber pathology parallels disease progression in Parkinson disease: a longitudinal study. Acta Neuropathol. 136, 501–503. doi: 10.1007/s00401-018-1876-1 29916036

[B194] Nouh C. D. Younes K. (2024). Diagnosis and management of progressive corticobasal syndrome. Curr. Treat Options Neurol. 26, 319–338. doi: 10.1007/s11940-024-00797-4 39886562 PMC11781596

[B195] Nysetvold E. Lopez L. N. Cogell A. N. Fryk H. Pace N. D. Taylor S. S. . (2024). Progressive Supranuclear palsy (PSP) disease progression, management, and healthcare resource utilization: a retrospective observational study in the US and Canada. Orphanet. J. Rare Dis. 19:215. doi: 10.1186/s13023-024-03168-z 38778404 PMC11112758

[B196] Ohtomo R. Tsuji S. Iwata A. (2016). Persistent pain as a non-motor symptom in corticobasal syndrome. J. Clin. Neurosci. 29, 35–37. doi: 10.1016/j.jocn.2015.11.025 26898576

[B197] Oliveira M. C. B. Ling H. Lees A. J. Holton J. L. De Pablo-Fernandez E. Warner T. T. (2019). Association of autonomic symptoms with disease progression and survival in progressive supranuclear palsy. J. Neurol. Neurosurg. Psychiatry 90, 555–561. doi: 10.1136/jnnp-2018-319374 30598430

[B198] Ory-Magne F. Pellaprat J. Harroch E. Galitzsky M. Rousseau V. Pavy-Le Traon A. . (2018). Abnormal pain perception in patients with multiple system atrophy. Parkinsonism Relat. Disord. 48, 28–33. doi: 10.1016/j.parkreldis.2017.12.001 29254664

[B199] Ozsancak C. Auzou P. Hannequin D. (2000). Dysarthria and orofacial apraxia in corticobasal degeneration. Mov. Disord. 15, 905–910. 11009198 10.1002/1531-8257(200009)15:5<905::aid-mds1022>3.0.co;2-d

[B200] Pál E. Fülöp K. Tóth P. Deli G. Pfund Z. Janszky J. . (2020). Small fiber neuropathy: clinicopathological correlations. Behav. Neurol. 2020:8796519. doi: 10.1155/2020/8796519 32399084 PMC7199601

[B201] Pang K. M. Park J. W. (2015). Masticatory muscle pain and progressive mouth opening limitation caused by amyotrophic lateral sclerosis: a case report. J. Oral Facial Pain Headache 29, 91–96. doi: 10.11607/ofph.1340 25635964

[B202] Paulsen J. S. Langbehn D. R. Stout J. C. Aylward E. Ross C. A. Nance M. . (2008). Detection of Huntington's disease decades before diagnosis: the Predict-HD study. J. Neurol. Neurosurg. Psychiatry 79, 874–880. doi: 10.1136/jnnp.2007.128728 18096682 PMC2569211

[B203] Pavlovic S. Stevic Z. Milovanovic B. Milicic B. Rakocevic-Stojanovic V. Lavrnic D. . (2010). Impairment of cardiac autonomic control in patients with amyotrophic lateral sclerosis. Amyotroph. Lateral Scler. 11, 272–276. doi: 10.3109/17482960903390855 20001491

[B204] Payan C. A. Viallet F. Landwehrmeyer B. G. Bonnet A. M. Borg M. Durif F. . (2011). Disease severity and progression in progressive supranuclear palsy and multiple system atrophy: validation of the NNIPPS–Parkinson Plus Scale. PLoS ONE 6:e22293. doi: 10.1371/journal.pone.0022293 21829612 PMC3150329

[B205] Peña-Zelayeta L. Delgado-Minjares K. M. Villegas-Rojas M. M. León-Arcia K. Santiago-Balmaseda A. Andrade-Guerrero J. . (2025). Redefining non-motor symptoms in Parkinson's disease. J. Pers. Med. 15:172. doi: 10.20944/preprints202503.1395.v1 40423044 PMC12112995

[B206] Perrotta A. Serpino C. Cormio C. Serrao M. Sandrini G. Pierelli F. . (2012). Abnormal spinal cord pain processing in Huntington's disease. The role of the diffuse noxious inhibitory control. Clin. Neurophysiol. 123, 1624–1630. doi: 10.1016/j.clinph.2012.01.012 22341978

[B207] Philips T. Rothstein J. D. (2014). Glial cells in amyotrophic lateral sclerosis. Exp. Neurol. 262(Pt B), 111–120. doi: 10.1016/j.expneurol.2014.05.015 24859452 PMC4241182

[B208] Piccione E. A. Sletten D. M. Staff N. P. Low P. A. (2015). Autonomic system and amyotrophic lateral sclerosis. Muscle Nerve. 51, 676–679. doi: 10.1002/mus.24457 25211238 PMC4362936

[B209] Pizarro-Galleguillos B. M. Kunert L. Brüggemann N. Prasuhn J. (2023). Neuroinflammation and mitochondrial dysfunction in Parkinson's disease: connecting neuroimaging with pathophysiology. Antioxidants 12:1411. doi: 10.3390/antiox12071411 37507950 PMC10375976

[B210] Pressnell Z. S. Neilson L. E. Tupone D. Pfeiffer R. F. Safarpour D. (2025). Thermoregulatory dysfunction in Parkinson's disease: mechanisms, implications, and therapeutic perspectives. Cells 14:1910. doi: 10.3390/cells14231910 41369399 PMC12691499

[B211] Provinciali L. Cangiotti A. Tulli D. Carboni V. Cinti S. (1994). Skin abnormalities and autonomic involvement in the early stage of amyotrophic lateral sclerosis. J. Neurol. Sci. 126, 54–61. doi: 10.1016/0022-510X(94)90094-9 7836947

[B212] Qian X. Yue L. Mellor D. Robbins N. M. Li W. Xiao S. (2022). Reduced peripheral nerve conduction velocity is associated with Alzheimer's disease: a cross-sectional study from China. Neuropsychiatr. Dis. Treat. 18, 231–242. doi: 10.2147/NDT.S349005 35177907 PMC8846612

[B213] Querol L. Devaux J. Rojas-Garcia R. Illa I. (2017). Autoantibodies in chronic inflammatory neuropathies: diagnostic and therapeutic implications. Nat. Rev. Neurol. 13, 533–547. doi: 10.1038/nrneurol.2017.84 28708133

[B214] Quinn C. Elman L. (2020). Amyotrophic lateral sclerosis and other motor neuron diseases. Continuum 26, 1323–1347. doi: 10.1212/CON.0000000000000911 33003004

[B215] Radicati F. G. Martinez Martin P. Fossati C. Chaudhuri K. R. Torti M. Rodriguez Blazquez C. . (2017). Non motor symptoms in progressive supranuclear palsy: prevalence and severity. NPJ Parkinsons Dis. 3:35. doi: 10.1038/s41531-017-0037-x 29238748 PMC5722941

[B216] Ramachandran A. Jose J. Gafoor V. A. Das S. Balaram N. (2022). Prevalence and risk factors of peripheral neuropathy in Parkinson's disease. Ann. Indian Acad. Neurol. 25, 1109–1115. doi: 10.4103/aian.aian_669_22 36911466 PMC9996500

[B217] Ramey G. D. Tang A. Phongpreecha T. Yang M. M. Woldemariam S. R. Oskotsky T. T. . (2024). Exposure to autoimmune disorders increases Alzheimer's disease risk in a multi-site electronic health record analysis. medRxiv. [preprint]. doi: 10.1101/2024.05.02.24306649 39999839 PMC11970322

[B218] Reddy K. Dieriks B. V. (2022). Multiple system atrophy: α-Synuclein strains at the neuron-oligodendrocyte crossroad. Mol Neurodegener. 17:77. doi: 10.1186/s13024-022-00579-z 36435784 PMC9701437

[B219] Reiss A. B. Arain H. A. Stecker M. M. Siegart N. M. Kasselman L. J. (2018). Amyloid toxicity in Alzheimer's disease. Rev. Neurosci. 29, 613–627. doi: 10.1515/revneuro-2017-0063 29447116

[B220] Ren Y. Liu W. Li Y. Sun B. Li Y. Yang F. . (2018). Cutaneous somatic and autonomic nerve TDP-43 deposition in amyotrophic lateral sclerosis. J. Neurol. 265, 1753–1763. doi: 10.1007/s00415-018-8897-5 29804146

[B221] Respondek G. Stamelou M. Kurz C. Ferguson L. W. Rajput A. Chiu W. Z. . (2014). The phenotypic spectrum of progressive supranuclear palsy: a retrospective multicenter study of 100 definite cases. Mov. Disord. 29, 1758–1766. doi: 10.1002/mds.26054 25370486

[B222] Rodolico C. Toscano A. De Luca G. Mazzeo A. Di Leo R. Baldari S. . (2001). Peripheral neuropathy as the presenting feature of multiple system atrophy. Clin. Auton. Res. 11, 119–121. doi: 10.1007/BF02322056 11570601

[B223] Rodríguez-Leyva I. Chi-Ahumada E. G. Carrizales J. Rodríguez-Violante M. Velázquez-Osuna S. Medina-Mier V. . (2016). Parkinson disease and progressive supranuclear palsy: protein expression in skin. Ann. Clin. Transl. Neurol. 3, 191–199. doi: 10.1002/acn3.285 27042679 PMC4774258

[B224] Rodriguez-Raecke R. Niemeier A. Ihle K. Ruether W. May A. (2009). Brain gray matter decrease in chronic pain is the consequence and not the cause of pain. J. Neurosci. 29, 13746–13750. doi: 10.1523/JNEUROSCI.3687-09.2009 19889986 PMC6666725

[B225] Rohmann R. Kühn E. Scherbaum R. Hilker L. Kools S. Scholz L. . (2021). Prevalence and characteristics of polyneuropathy in atypical parkinsonian syndromes: an explorative study. Brain Sci. 11:879. doi: 10.3390/brainsci11070879 34209067 PMC8301815

[B226] Rubio M. A. Herrando-Grabulosa M. Navarro X. (2022). Sensory involvement in amyotrophic lateral sclerosis. Int. J. Mol. Sci. 23:15521. doi: 10.3390/ijms232415521 36555161 PMC9779879

[B227] Ruiz-Soto M. Riancho J. Tapia O. Lafarga M. Berciano M. T. (2020). Satellite glial cells of the dorsal root ganglion: a new “Guest/Physiopathological Target” in ALS. Front. Aging Neurosci. 12:595751. doi: 10.3389/fnagi.2020.595751 33240079 PMC7680735

[B228] Safieh M. Liraz O. Ovadia M. Michaelson D. (2024). The role of impaired receptor trafficking in mediating the pathological effects of APOE4 in Alzheimer's disease. J. Alzheimers Dis. 97, 753–775. doi: 10.3233/JAD-230514 38217595 PMC10894586

[B229] Samolis A. Troupis T. Politis C. Pantazis N. Triantafyllou G. Tsakotos G. . (2025). Intraepidermal nerve fiber density as an indicator of neuropathy predisposition: a systematic review with meta-analysis. Diagnostics 15:1311. doi: 10.3390/diagnostics15111311 40506883 PMC12154223

[B230] Sarto J. Esteller-Gauxax D. Guillén N. Falgàs N. Borrego-Écija S. Massons M. . (2025). Accuracy and clinical applicability of plasma tau 181 and 217 for Alzheimer's disease diagnosis in a memory clinic cohort. J. Neurol. 272:160. doi: 10.1007/s00415-025-12897-5 39849125

[B231] Sassone J. Colciago C. Cislaghi G. Silani V. Ciammola A. (2009). Huntington's disease: the current state of research with peripheral tissues. Exp. Neurol. 219, 385–397. doi: 10.1016/j.expneurol.2009.05.012 19460373

[B232] Sassone J. Taiana M. Lombardi R. Porretta-Serapiglia C. Freschi M. Bonanno S. . (2016). ALS mouse model SOD1G93A displays early pathology of sensory small fibers associated to accumulation of a neurotoxic splice variant of peripherin. Hum. Mol. Genet. 25, 1588–1599. doi: 10.1093/hmg/ddw035 26908600

[B233] Scherder E. Statema M. (2010). Huntington's disease. Lancet 376:1464. doi: 10.1016/S0140-6736(10)61990-3 21036270

[B234] Schrag A. Selai C. Mathias C. Low P. Hobart J. Brady N. . (2007). Measuring health-related quality of life in MSA: the MSA-QoL. Mov. Disord. 22, 2332–2338. doi: 10.1002/mds.21649 17914730

[B235] Schrag A. Sheikh S. Quinn N. P. Lees A. J. Selai C. Mathias C. . (2010). A comparison of depression, anxiety, and health status in patients with progressive supranuclear palsy and multiple system atrophy. Mov. Disord. 25, 1077–10781. doi: 10.1002/mds.22794 20535826

[B236] Schreml S. Kaiser E. Landthaler M. Szeimies R. M. Babilas P. (2010). Amyloid in skin and brain: what's the link? Exp. Dermatol. 19, 953–957. doi: 10.1111/j.1600-0625.2010.01166.x 20807234

[B237] Schulte J. Littleton J. T. (2011). The biological function of the Huntingtin protein and its relevance to Huntington's Disease pathology. Curr. Trends Neurol. 5, 65–78. 22180703 PMC3237673

[B238] Seki S. Kitaoka Y. Kawata S. Nishiura A. Uchihashi T. Hiraoka S. I. . (2023). Characteristics of sensory neuron dysfunction in amyotrophic lateral sclerosis (ALS): potential for ALS therapy. Biomedicines 11:2967. doi: 10.3390/biomedicines11112967 38001967 PMC10669304

[B239] Sempere-Bigorra M. Julián-Rochina I. Cauli O. (2021). Differences and similarities in neuropathy in type 1 and 2 diabetes: a systematic review. J. Pers. Med. 11:230. doi: 10.3390/jpm11030230 33810048 PMC8004786

[B240] Shi Y. Zhang W. Yang Y. Murzin A. G. Falcon B. Kotecha A. . (2021). Structure-based classification of tauopathies. Nature 598, 359–363. doi: 10.1038/s41586-021-03911-7 34588692 PMC7611841

[B241] Shim K. H. Kang M. J. Youn Y. C. An S. S. A. Kim S. (2022). Alpha-synuclein: a pathological factor with Aβ and tau and biomarker in Alzheimer's disease. Alzheimers Res. Ther. 14:201. doi: 10.1186/s13195-022-01150-0 36587215 PMC9805257

[B242] Shojaie A. Al Khleifat A. Opie-Martin S. Sarraf P. Al-Chalabi A. (2024). Non-motor symptoms in amyotrophic lateral sclerosis. Amyotroph. Lateral Scler. Frontotemporal. Degener. 25, 61–66. doi: 10.1080/21678421.2023.2263868 37798838 PMC11090076

[B243] Sprenger G. P. van Velzen M. Achterberg W. P. Roos R. A. C. de Bot S. T. (2026). Pain processing and pain assessment in Huntington's disease: study protocol of the Huntington's disease - PAIN study. PLoS ONE. 21:e0346039. doi: 10.1371/journal.pone.0346039 41961860 PMC13068213

[B244] Stamelou M. Dohmann H. Brebermann J. Boura E. Oertel W. H. Höglinger G. . (2012). Clinical pain and experimental pain sensitivity in progressive supranuclear palsy. Parkinsonism Rela. Disord. 18, 606–618. doi: 10.1016/j.parkreldis.2011.11.010 22130148

[B245] Steele J. C. (1972). Progressive supranuclear palsy. Brain 95, 693–704. 4647151

[B246] Sun J. Wang Y. Zhang X. Zhu S. He H. (2020). Prevalence of peripheral neuropathy in patients with diabetes: a systematic review and meta-analysis. Prim. Care Diabetes 14, 435–444. doi: 10.1016/j.pcd.2019.12.005 31917119

[B247] Swallow D. M. A. Zheng C. S. Counsell C. E. (2022). Systematic review of prevalence studies of progressive supranuclear palsy and corticobasal syndrome. Mov. Disord. Clin. Pract. 9, 604–613. doi: 10.1002/mdc3.13489 35844273 PMC9274340

[B248] Taams N. E. Drenthen J. Hanewinckel R. Ikram M. A. van Doorn P. A. (2023). Age-related changes in neurologic examination and sensory nerve amplitude in the general population: aging of the peripheral nervous system. Neurology 101, e1351–e8. doi: 10.1212/WNL.0000000000207665 37541844 PMC10558170

[B249] Taga A. Schito P. Trapasso M. C. Zinno L. Pavesi G. (2019). Pain at the onset of amyotrophic lateral sclerosis: a cross-sectional study. Clin. Neurol. Neurosurg. 186:105540. doi: 10.1016/j.clineuro.2019.105540 31634749

[B250] Tai Y. C. Lin C. H. (2020). An overview of pain in Parkinson's disease. Clin. Park Relat. Disord. 2, 1–8. doi: 10.1016/j.prdoa.2019.11.004 34316612 PMC8302194

[B251] Tanaka H. Martinez-Valbuena I. Forrest S. L. Couto B. Reyes N. G. Morales-Rivero A. . (2024). Distinct involvement of the cranial and spinal nerves in progressive supranuclear palsy. Brain. 147, 1399–411. doi: 10.1093/brain/awad381 37972275 PMC10994524

[B252] Testa D. Monza D. Ferrarini M. Soliveri P. Girotti F. Filippini G. (2001). Comparison of natural histories of progressive supranuclear palsy and multiple system atrophy. Neurol. Sci. 22, 247–251. doi: 10.1007/s100720100021 11731878

[B253] Teunissen C. E. Chiu M. J. Yang C. C. Yang S. Y. Scheltens P. Zetterberg H. . (2018). Plasma amyloid-β (Aβ42) correlates with cerebrospinal fluid Aβ42 in Alzheimer's disease. J. Alzheimers Dis. 62, 1857–1863. doi: 10.3233/JAD-170784 29614646

[B254] The Huntington's Disease Collaborative Research Group (1993). A novel gene containing a trinucleotide repeat that is expanded and unstable on Huntington's disease chromosomes. Cell 72, 971–983. doi: 10.1016/0092-8674(93)90585-E 8458085

[B255] Thomas H. A. Goudman L. DiMarzio M. Barron G. Pilitsis J. G. (2024). Prevalence of pain phenotypes and co-morbidities of chronic pain in Parkinson's disease. Clin. Neurol. Neurosurg. 246:108563. doi: 10.1016/j.clineuro.2024.108563 39299006

[B256] Thomas S. Enders J. Kaiser A. Rovenstine L. Heslop L. Hauser W. . (2023). Abnormal intraepidermal nerve fiber density in disease: a scoping review. Front. Neurol. 14:1161077. doi: 10.3389/fneur.2023.1161077 37153658 PMC10157176

[B501] Thongchuam Y. Panyakaew P. Bhidayasiri R. (2020). Orofacial dystonia and asssociated bulbar symptoms in multiple system atrophy: a blinded video analysis. J. Neurol. Sci. 417:116992. doi: 10.1016/j.jns.2020.116992 32622521

[B257] Tian J. Jones G. Lin X. Zhou Y. King A. Vickers J. . (2023). Association between chronic pain and risk of incident dementia: findings from a prospective cohort. BMC Med. 21:169. doi: 10.1186/s12916-023-02875-x 37143042 PMC10161483

[B258] Tison F. Wenning G. K. Volonte M. A. Poewe W. R. Henry P. Quinn N. P. (1996). Pain in multiple system atrophy. J. Neurol. 243, 153–156. doi: 10.1007/BF02444007 8750553

[B259] Toth C. Brown M. S. Furtado S. Suchowersky O. Zochodne D. (2008). Neuropathy as a potential complication of levodopa use in Parkinson's disease. Mov. Disord. 23, 1850–1859. doi: 10.1002/mds.22137 18785232

[B260] Trelle A. N. Young C. B. Vossler H. Ramos Benitez J. Cody K. A. Mendiola J. H. . (2025). Plasma Aβ/Aβ is sensitive to early cerebral amyloid accumulation and predicts risk of cognitive decline across the Alzheimer's disease spectrum. Alzheimer Dement. 21:e14442. doi: 10.1002/alz.14442 39713875 PMC11848181

[B261] Tsai R. M. Boxer A. L. (2014). Treatment of frontotemporal dementia. Curr. Treat. Options Neurol. 16:319. doi: 10.1007/s11940-014-0319-0 25238733 PMC4920050

[B262] Underwood M. Bonas S. Dale M. (2017). Huntington's disease: prevalence and psychological indicators of pain. Mov. Disord. Clin. Pract. 4, 198–204. doi: 10.1002/mdc3.12376 30363479 PMC6174505

[B263] Unti E. Mazzucchi S. Calabrese R. Palermo G. Del Prete E. Bonuccelli U. . (2019). Botulinum toxin for the treatment of dystonia and pain in corticobasal syndrome. Brain Behav. 9:e01182. doi: 10.1002/brb3.1182 31074111 PMC6576166

[B264] Urso D. Giannoni-Luza S. Brayne C. Ray N. Logroscino G. (2025). Incidence and prevalence of frontotemporal dementia: a systematic review and meta-analysis. JAMA Neurol. 82, 1144–1152. doi: 10.1001/jamaneurol.2025.3307 40920400 PMC12418226

[B265] Vacchi E. Lazzarini E. Pinton S. Chiaro G. Disanto G. Marchi F. . (2022). Tau protein quantification in skin biopsies differentiates tauopathies from alpha-synucleinopathies. Brain 145, 2755–2768. doi: 10.1093/brain/awac161 35485527

[B266] Vacchi E. Senese C. Chiaro G. Disanto G. Pinton S. Morandi S. . (2021). Alpha-synuclein oligomers and small nerve fiber pathology in skin are potential biomarkers of Parkinson's disease. NPJ Parkinsons Dis. 7:119. doi: 10.1038/s41531-021-00262-y 34930911 PMC8688481

[B267] van Kooten J. Delwel S. Binnekade T. T. Smalbrugge M. van der Wouden J. C. Perez R. S. . (2015). Pain in dementia: prevalence and associated factors: protocol of a multidisciplinary study. BMC Geriatr. 15:29. doi: 10.1186/s12877-015-0025-0 25879681 PMC4436741

[B268] Vela L. Cano-de-la-Cuerda R. Fil A. Muñoz-Hellín E. Ortíz-Gutiérrez R. Macías-Macías Y. . (2012). Thermal and mechanical pain thresholds in patients with fluctuating Parkinson's disease. Parkinsonism Relat. Disord. 18, 953–957. doi: 10.1016/j.parkreldis.2012.04.031 22658653

[B269] Verdú E. Ceballos D. Vilches J. J. Navarro X. (2000). Influence of aging on peripheral nerve function and regeneration. J. Peripher. Nerv. Syst. 5, 191–208. doi: 10.1111/j.1529-8027.2000.00026.x 11151980

[B270] Verhoeff M. C. Koutris M. Tambach S. Eikenboom D. de Vries R. Berendse H. W. . (2022). Orofacial pain and dysfunction in patients with Parkinson's disease: a scoping review. Eur. J. Pain. 26, 2036–2059. doi: 10.1002/ejp.2031 36063442 PMC9826258

[B271] Verschueren A. (2017). Motor neuropathies and lower motor neuron syndromes. Rev. Neurol. 173, 320–325. doi: 10.1016/j.neurol.2017.03.018 28434507

[B272] Voitenkov V. B. Komancev V. N. Skripchenko N. V. Grigoryev S. G. Klimkin A. V. Aksenova A. I. (2017). [Age-related changes of peripheral nerve system and muscles of the limbs in healthy persons]. Adv. Gerontol. 30, 78–83. 28557394

[B273] Vonsattel J. P. Myers R. H. Stevens T. J. Ferrante R. J. Bird E. D. Richardson E. P. (1985). Jr. Neuropathological classification of Huntington's disease. J. Neuropathol. Exp. Neurol. 44, 559–577. doi: 10.1097/00005072-198511000-00003 2932539

[B274] Wang K. Liu H. (2021). Association between widespread pain and dementia, Alzheimer's disease and stroke: a cohort study from the Framingham Heart Study. Reg. Anesth. Pain Med. 46, 879–885. doi: 10.1136/rapm-2021-102733 34400574

[B275] Wang Y. Xie M. Xu D. Wang Y. Wang H. (2025). Autonomic dysfunction in progressive supranuclear palsy: a retrospective study. Clin. Park. Relat. Disord. 12:100310. doi: 10.1016/j.prdoa.2025.100310 40124981 PMC11927701

[B276] Watanabe H. Riku Y. Hara K. Kawabata K. Nakamura T. Ito M. . (2018). Clinical and imaging features of multiple system atrophy: challenges for an early and clinically definitive diagnosis. J. Mov. Disord. 11, 107–120. doi: 10.14802/jmd.18020 30086614 PMC6182302

[B277] Watanabe H. Shima S. Mizutani Y. Ueda A. Ito M. (2023). Multiple system atrophy: advances in diagnosis and therapy. J. Mov. Disord. 16, 13–21. doi: 10.14802/jmd.22082 36537066 PMC9978260

[B278] Weaver J. Kim K. W. Carey B. T. Langsdorf J. A. Chin R. L. (2018). Gender differences in skin biopsy findings in small fiber neuropathy: a retrospective chart review. J. Clin. Neuromuscul. Dis. 20, 69–75. doi: 10.1097/CND.0000000000000228 30439752

[B279] Webster J. M. Yang Y. T. Miller A. T. Zane A. Scholz K. Stone W. J. . (2025). Tau, amyloid-β and α-synuclein co-pathologies synergistically enhance neuroinflammation and neuropathology. bioRxiv. [preprint]. doi: 10.1101/2024.10.13.618101 41747969

[B280] Weis J. Katona I. Müller-Newen G. Sommer C. Necula G. Hendrich C. . (2011). Small-fiber neuropathy in patients with ALS. Neurology 76, 2024–2029. doi: 10.1212/WNL.0b013e31821e553a 21646630

[B281] Wen S. Elias P. M. Wakefield J. S. Mauro T. M. Man M. Q. (2022). The link between cutaneous inflammation and cognitive impairment. J. Eur. Acad. Dermatol. Venereol. 36, 1705–1712. doi: 10.1111/jdv.18360 35748522 PMC9481668

[B282] Wen Y. Yang Q. Jiao B. Zhang W. Lin J. Zhu Y. . (2023). Clinical features of progressive supranuclear palsy. Front. Aging Neurosci. 15:1229491. doi: 10.3389/fnagi.2023.1229491 37711994 PMC10498458

[B283] Whitlock E. L. Diaz-Ramirez L. G. Glymour M. M. Boscardin W. J. Covinsky K. E. Smith A. K. (2017). Association between persistent pain and memory decline and dementia in a longitudinal cohort of elders. JAMA Intern. Med. 177, 1146–1153. doi: 10.1001/jamainternmed.2017.1622 28586818 PMC5588896

[B284] Whitney K. Song W. M. Sharma A. Dangoor D. K. Farrell K. Krassner M. M. . (2024). Single-cell transcriptomic and neuropathologic analysis reveals dysregulation of the integrated stress response in progressive supranuclear palsy. Acta Neuropathol. 148:80. doi: 10.1007/s00401-024-02823-w 39648200 PMC11625691

[B285] Windebank A. J. Blexrud M. D. Dyck P. J. Daube J. R. Karnes J. L. (1990). The syndrome of acute sensory neuropathy: clinical features and electrophysiologic and pathologic changes. Neurology 40, 584–591. doi: 10.1212/WNL.40.4.584 2157173

[B286] Woerman A. L. Watts J. C. Aoyagi A. Giles K. Middleton L. T. Prusiner S. B. (2018). α-synuclein: multiple system atrophy prions. Cold Spring Harb. Perspect. Med. 8:a024588. doi: 10.1101/cshperspect.a024588 28213437 PMC5561534

[B287] Woo K. A. Shin J. Y. Kim H. Ahn J. Jeon B. Lee J. Y. (2022). Peripapillary retinal nerve fiber layer thinning in patients with progressive supranuclear palsy. J. Neurol. 269, 3216–3225. doi: 10.1007/s00415-021-10936-5 34921616 PMC9120117

[B288] Xie C. Chen J. Gan R. Gao Y. Wang L. Ma G. . (2025). Patients with small fiber neuropathy exhibit more pronounced non-motor symptoms in Parkinson's disease. J. Parkinsons Dis. 15, 1442–1451. doi: 10.1177/1877718X251371538 40891687 PMC13347533

[B289] Xu B. Lei X. Yang Y. Yu J. Chen J. Xu Z. . (2025). Peripheral proteinopathy in neurodegenerative diseases. Transl. Neurodegener. 14:2. doi: 10.1186/s40035-024-00461-6 39819742 PMC11737199

[B290] Xu L. Pu J. (2016). Alpha-synuclein in Parkinson's disease: from pathogenetic dysfunction to potential clinical application. Parkinsons Dis. 2016:1720621. doi: 10.1155/2016/1720621 27610264 PMC5005546

[B291] Yang J. Ogasa T. Ohta Y. Abe K. Wu J. (2010). Decline of human tactile angle discrimination in patients with mild cognitive impairment and Alzheimer's disease. J. Alzheimers Dis. 22, 225–234. doi: 10.3233/JAD-2010-100723 20847416

[B505] Yao K. Wang S. Xu Z. Fan Z. Chen Z. Jia P. . (2025). Mechanisms of comorbidity between Alzheimer's disease and pain. Alzheimers Dement. 21:e14605. doi: 10.1002/alz.14605 PMC11852355 39998175

[B292] Yegambaram M. Manivannan B. Beach T. G. Halden R. U. (2015). Role of environmental contaminants in the etiology of Alzheimer's disease: a review. Curr. Alzheimer Res. 12, 116–146. doi: 10.2174/1567205012666150204121719 25654508 PMC4428475

[B293] Zafar S. Yaddanapudi S. S. (2025). Parkinson Disease. StatPearls. Treasure Island, FL: StatPearls Publishing. 29261972

[B294] Zambelis T. Karandreas N. Tzavellas E. Kokotis P. Liappas J. (2005). Large and small fiber neuropathy in chronic alcohol-dependent subjects. J. Peripher. Nerv. Syst. 10, 375–381. doi: 10.1111/j.1085-9489.2005.00050.x 16279987

[B295] Zhao W. Zhao L. Chang X. Lu X. Tu Y. (2023). Elevated dementia risk, cognitive decline, and hippocampal atrophy in multisite chronic pain. Proc. Natl. Acad. Sci. USA 120:e2215192120. doi: 10.1073/pnas.2215192120 36802440 PMC9992778

